# Lightweight pear detection in unstructured orchards via selective information propagation

**DOI:** 10.3389/fpls.2026.1835001

**Published:** 2026-07-13

**Authors:** Bingyu Cao, Mingqi Kan, Wei Chen, Yingchao Wang, Peng Zhou, Zhikai Yang

**Affiliations:** 1 Information Science and Engineering, Xinjiang College of Science & Technology, Korla, Xinjiang, China; 2 Artificial Intelligence and Data Science, Hebei University of Technology, Tianjin, China; 3 School of Artificial Intelligence, YanShan University, Qinhuangdao, Hebei, China

**Keywords:** agricultural robotics, lightweight object detection, pear detection, state-space modeling, unstructured orchard

## Abstract

Accurate pear detection in unstructured orchards is important for robotic harvesting and orchard perception. However, pear detection poses compound challenges that differ from those in more chromatically distinctive fruits: mature pears share yellow-green hues with surrounding foliage, their near-spherical geometry offers limited contour priors, and they typically grow in tight spur clusters where mutual boundary occlusion occurs even without branch interference. Under these coupled degradations, lightweight detectors tend to lose accuracy and become difficult to deploy on embedded agricultural platforms. To address this issue, we propose a lightweight pear detection framework guided by the principle of selective information propagation—the idea that, under tight computational budgets, how information is routed at each stage matters more than overall network capacity. The framework instantiates this principle along four stages of the detection pipeline through dedicated modules for efficient global–local context modeling, input-adaptive feature transformation, detail-preserving multiscale fusion, and an adaptive IoU loss tailored for small and occluded fruits. On the self-built Orchard Pear dataset, the proposed method achieves 95.2% mAP@50 and 54.6% mAP@50:95 with only 2.56 M parameters and 5.60 GFLOPs. Consistent improvements are also observed on the public Minne Apple and Mango datasets. Deployment experiments on embedded platforms further show that the proposed method supports real-time inference for agricultural robotic applications. These results suggest that selective feature representation, fusion, and optimization benefit lightweight fruit detection in complex orchard scenes.

## 1 Introduction

Accurate fruit detection underpins robotic harvesting and precision orchard management. Among fruit categories, pear detection in unstructured orchards presents a particular combination of difficulties that extend beyond the generic occlusion and illumination issues shared by most agricultural scenes. First, commercially important cultivars such as Korla fragrant pear (*Pyrus sinkiangensis* Yü) retain a yellow-green surface color throughout ripening, producing low chromatic contrast against canopy foliage that weakens the color cues general-purpose detectors rely on. Second, pears tend to grow in compact clusters along short spurs, so adjacent instances frequently share occluding boundaries even when no branches or leaves are involved—a pattern that differs from the branch-mediated occlusion more typical of apples or citrus. Third, the near-rotational symmetry of pear contours deprives detectors of the distinctive shape priors that aid recognition of more angular fruits, making partial visibility especially difficult to resolve. These fruit-specific factors, rather than generic environmental variation, motivate a detection framework tailored to pear morphology under lightweight constraints (Liu et al., 2024a).

For orchard perception under embedded constraints, lightweight one-stage detectors of the YOLO family have become the standard choice, owing to their favorable balance between accuracy and inference speed (Yang et al., 2024; Wang et al., 2024a; Wang et al., 2024c). Recent pear- and fruit-detection studies along this line have pursued stronger backbone extraction, enhanced multi-scale fusion, and attention-based refinement (Ma et al., 2025; Wang et al., 2024b; Tang et al., 2025; Wang et al., 2025b). These efforts largely transplant general detection improvements into the orchard setting, yet do not explicitly address the pear-specific morphological factors described above. Related work in agricultural vision has also sought gains beyond network architecture. At the feature level, metaheuristic selection can prune redundant handcrafted descriptors and improve plant-disease classification (Xie et al., 2023); at the domain level, transfer learning across mixed subdomains improves diagnosis between poorly correlated species (Yan et al., 2023). Both operate on leaf-level disease classification rather than in-field fruit detection, yet they raise the same questions our design addresses: which features to retain, and how learned representations transfer across domains.

Despite these advances, lightweight detectors still show measurable performance degradation in orchard scenes with heavy occlusion, dense small objects, and unstable illumination (Xiao et al., 2025). Our preliminary analysis on the Orchard Pear dataset quantifies this gap directly: the YOLOv11n baseline reaches 47.5% mAP@50:95 on the full test set, but drops to 28.3% on the small-scale subset (a relative reduction of 40.4%), 39.8% on the heavily occluded subset, and 41.2% under strong-illumination imagery (**Table 1**). Similar scenario-dependent degradation has been reported in recent pear detection studies, where lightweight YOLO variants lose 5–15 percentage points when moving from curated to heavily occluded scenes (Ma et al., 2025; Liu et al., 2024a). The underlying cause is that different orchard-specific degradations impair different stages of the detection pipeline: repeated downsampling erodes fine structural details for small and partially occluded fruits; occlusion and illumination variation weaken local appearance cues; dense small instances tend to lose structural details during feature fusion; and small or partially occluded targets often provide weak regression signals during training (Jocher and Qiu, 2024).

**Table 1:** Scenario-specific performance analysis on the Orchard Pear dataset.

Model configuration	Overall mAP@50:95	Branch-leaf occlusion	Dense small-scale	Intense illumination
YOLOv11n Baseline	47.50	39.80	28.30	41.20
+ CSP-SGLFE	51.50	43.10	32.50	46.80
+ SGEAE	52.10	46.20	33.80	44.50
+ CSDU+MSCRB	51.40	42.60	35.90	43.70
+ SOA-IoU	49.15	43.80	31.80	42.30
Full Model (Ours)	54.60	48.50	38.20	49.10

This stage-by-stage pattern is what shapes our design. The three subsets do not weaken by a common factor: the small-scale subset falls to 28.3% (a 40.4% relative drop), whereas the occluded and strongly lit subsets hold at 39.8% and 41.2%. A single, global shortage of capacity—one that more layers or channels would close—would not produce so uneven a profile; the deficit reads instead as the sum of several local mismatches, each at the stage identified above. Under a fixed parameter budget, scaling the network is therefore the wrong lever. What matters is the fit between each stage and the input it receives: whether the information arriving there is processed in a way suited to it. We refer to this as selective information propagation.

The four failure points each call for a change at the stage where they arise. In the backbone, the input decides how much weight contextual evidence carries relative to local cues, replacing the static residual weight that would otherwise fix this balance in advance; and regions of differing difficulty are routed to separate transformation paths instead of a single shared one—two responses aimed at the appearance cues that occlusion and lighting erode. The neck is the stage where plain top-down interpolation smears the thin boundaries between clustered fruits, so we rebuild the upsampling step to keep those boundaries through fusion. During training, each sample’s gradient is scaled by its own difficulty, preventing the few hard fruits from being averaged away by the easy majority. Section 2 develops these as four dedicated modules (Wang et al., 2019; Fu et al., 2025).

A framework built around pear-specific morphology carries an inherent risk: its gains may reflect overfitting to a single fruit category rather than transferable detection behavior. To examine this, we evaluated the method not only on a self-built Orchard Pear dataset but also on two public datasets chosen to stress the design in two complementary ways—Minne Apple, dominated by dense small instances, and Mango, characterized by low foreground– background contrast. Each dataset reproduces one of the core difficulties that motivate the design, namely small-scale density and chromatic camouflage, so that consistent gains on both would indicate that the modules capture properties generalizing beyond pear orchards. Deployment experiments on embedded platforms further assessed whether the method meets the latency and power budgets required for field robotic use.

The main contributions of this work are as follows:

- We propose a lightweight pear detection framework for unstructured orchards based on selective information propagation.
- The framework improves context modeling, adaptive feature transformation, multiscale fusion, and bounding-box regression under lightweight constraints.
- Experiments on one self-built dataset, two public datasets, and multiple embedded platforms demonstrate improved accuracy, cross-dataset generalization, and real-time deployment capability.
- Furthermore, to facilitate future research, our source code, trained models, and implementation details have been made publicly available at https://github.com/DynaVLA/LiFoSEA.

## 2 Materials and methods

### 2.1 Overall architecture overview

The four modules described below share a common computational form, which we state once before turning to each in detail. Consider a stage that maps an input feature *x* to an output through a set of candidate transformations ${\left\{{\text{Φ}}_{i}(x)\right\}}_{i=1}^{N}$, whose output is given by Equation 1:

(1) $$T(x)=\overset{N}{\underset{i=1}{\sum }}g_{i}(x;\theta )\cdot {\text{Φ}}_{i}(x),$$

where *g_i_*(*x*;*θ*) is a weighting function parameterized by *θ*. Propagation is *non-selective* when *g_i_*is independent of *x*, as in a fixed residual weight, a uniform branch average, or an unweighted loss aggregation. It is *selective* when *g_i_*varies with the input content or with per-sample statistics. The four modules differ only in the form *g_i_*takes and the stage at which it acts: in CSP-SGLFE it is an input-dependent gate over context (Section 2.2); in SGEAE a sparse Top-*K* router over experts (Section 2.3); in CSDU and MSCRB an allocation toward detail-preserving reorganization (Section 2.4); and in SOA-IoU a scale and occlusion-aware sample weight (Section 2.5).

**Figure 1:**
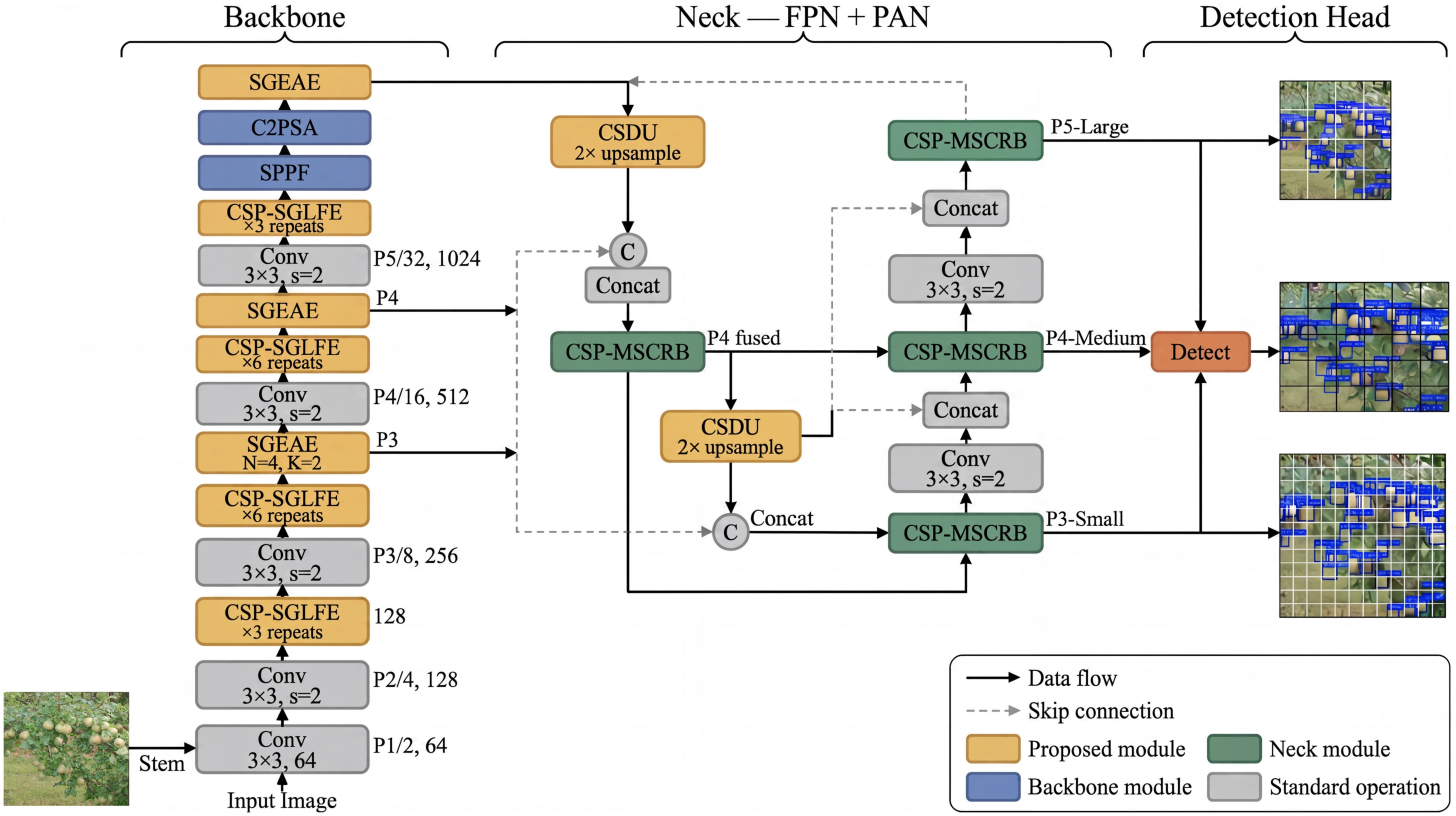
Overall architecture of the proposed pear detection framework. The backbone (left) follows the standard FPN convention: Stage 1, Stage 2, and Stage 3 produce the pyramid features P3/8, P4/16, and P5/32, respectively. CSPSGLFE is inserted at each stage for selective context propagation, and SGEAE (with *N* = 4, *K* = 2) is applied to the three pyramid outputs for input-adaptive feature transformation. The neck (middle) follows an FPN+PAN topology, where CSDU handles 2× top-down upsampling and CSP-MSCRB performs multi-scale fusion. The detection head (right) is kept identical to YOLOv11n and outputs {P3-Small, P4-Medium, P5-Large} predictions. Solid arrows denote forward data flow; dashed arrows denote skip connections. Tensor shapes beside each Conv block indicate the pyramid level and channel count.

To improve pear detection in unstructured orchards under limited computational budgets, we build the proposed method on YOLOv11n and enhance the baseline at the levels of backbone representation, neck fusion, and regression optimization. As shown in **Figure 1**, the framework contains four components: CSP-SGLFE for context enhancement in the backbone, SGEAE for adaptive feature transformation, CSDU and MSCRB for lightweight multi-scale fusion in the neck, and SOA-IoU for bounding-box regression during training.

**Figure 2:**
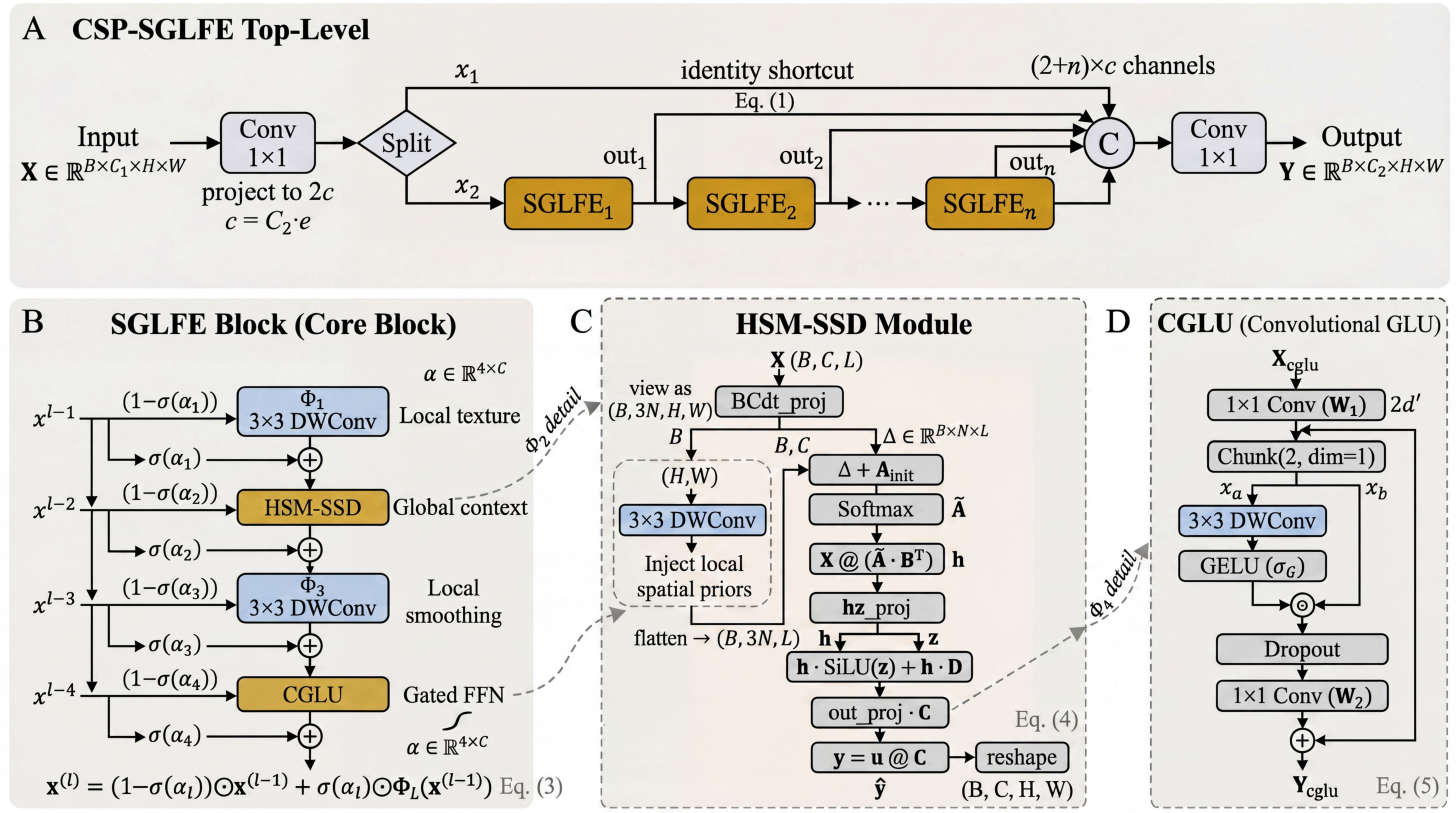
Architecture of CSP-SGLFE. Panel **(A)** shows the top-level cross-stage partial topology: the input $X\in {\mathbb{R}}^{B\times C_{1}\times H\times W}$ is projected to 2*c* channels (with *c* = *C*_2_ · *e*) via 1×1 convolution and split into *x*_1_ (identity shortcut) and *x*_2_, which is sequentially refined by *n* cascaded SGLFE blocks, followed by channel concatenation and 1×1 projection to $Y\in {\mathbb{R}}^{B\times C_{2}\times H\times W}$ (Equation 2). Panel **(B)** details the SGLFE block, a four-stage adaptive residual structure controlled by learnable scalings $\alpha \in {\mathbb{R}}^{4\times C}$: ${\text{Φ}}_{1}/{\bar{\text{Φ}}}_{3}$ are 3×3 depthwise convolutions for local texture and local smoothing, Φ_2_ is the HSM-SSD module for global context, and Φ_4_ is CGLU for gated feed-forward modulation (Equation 4). Panel **(C)** shows HSMSSD, where Δ, *B*, and *C* are projected and a 2D depthwise convolution injects local spatial priors before the state-space recurrence (Equation 5); its complexity is *O*(*C* · *N* · *L*) with *N* = 64 ≪ *L*. Panel D shows the CGLU gating pathway (Equation 6).

**Figure 3:**
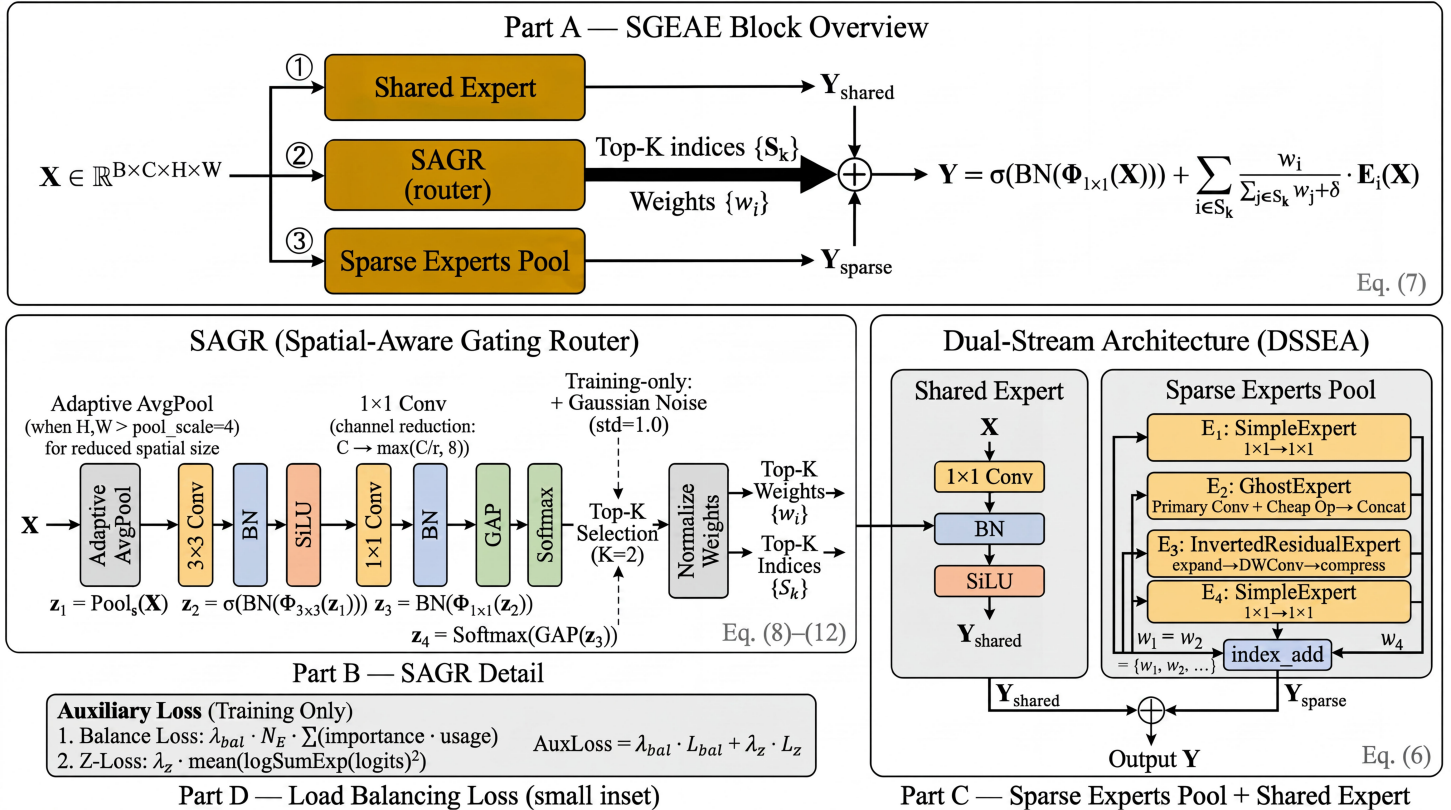
Architecture of SGEAE. Panel **(A)** shows the block overview: the input $X\in {\mathbb{R}}^{B\times C\times H\times W}$ is dispatched in parallel to (i) a Shared Expert producing *Y*_shared_, (ii) SAGR producing the Top-*K* indices {*S_k_*} and normalized weights {*w_i_*}, and (iii) the Sparse Experts Pool producing *Y*_sparse_; the two streams are added to yield *Y* (Equation 8). Panel **(B)** details the SAGR pipeline: adaptive average pooling, 3×3 convolution, BN, SiLU, 1×1 channel-mapping convolution, BN, GAP, and Softmax generate the routing logits *z*_4_, from which Top-*K* selection and weight normalization are performed (Equations 9-13); Gaussian noise (std=1.0) is injected into the logits only during training. Panel **(C)** shows the Dual-Stream Architecture (DSSEA): the heterogeneous expert pool comprises four experts {*E*_1_*, E*_2_*, E*_3_*, E*_4_}—two SimpleExperts, one GhostExpert, and one InvertedResidualExpert—and only the Top-*K* = 2 selected experts contribute to *Y*_sparse_ via an index-add operation (Equation 7). Panel D summarizes the auxiliary load-balancing and z-loss used only during training.

**Figure 4:**
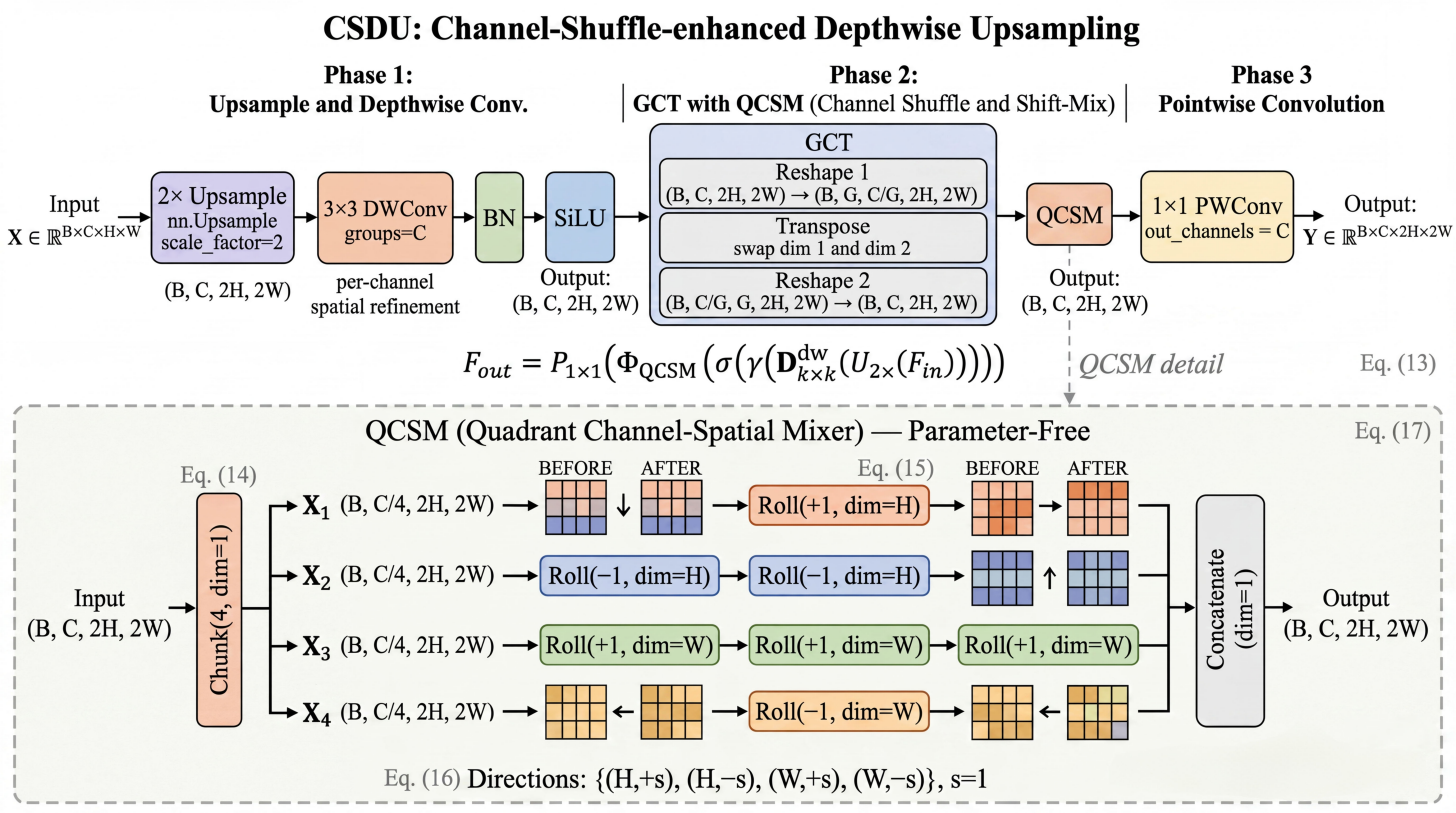
Structure of CSDU. Phase 1 upsamples $X\in {\mathbb{R}}^{B\times C\times H\times W}$ to (B*, C*, 2*H*, 2*W*) via 2× nearest-neighbor interpolation followed by a 3×3 depthwise convolution with groups=*C*. Phase 2 performs parameter-free channel-spatial mixing via GCT followed by QCSM. In the QCSM sub-panel, the four rows correspond to the channel partitions {*X*^(1)^*, X*^(2)^*, X*^(3)^*, X*^(4)^} produced by S^4^*^C^*(·) (Equation 15); within each row, the left grid is the input tile (BEFORE), the middle block denotes the cyclic-shift operator *T_di_, δ_i_*(·) applied once with step *s* = 1 (Equation 16), and the right grid is the output tile (AFTER); the four shift directions [(*H*, +*s*), (*H*, −*s*), (*W*, +*s*), (*W*, −*s*)] are listed in Equation 17. The concatenation of the four shifted tiles yields Φ_QCSM_(*X*) (Equation 18). Phase 3 projects the mixed feature back to the target channel dimension *C* via a 1×1 pointwise convolution, giving the output $Y=F_{\text{out}}\in {\mathbb{R}}^{B\times C\times 2H\times 2W}$.

**Figure 5:**
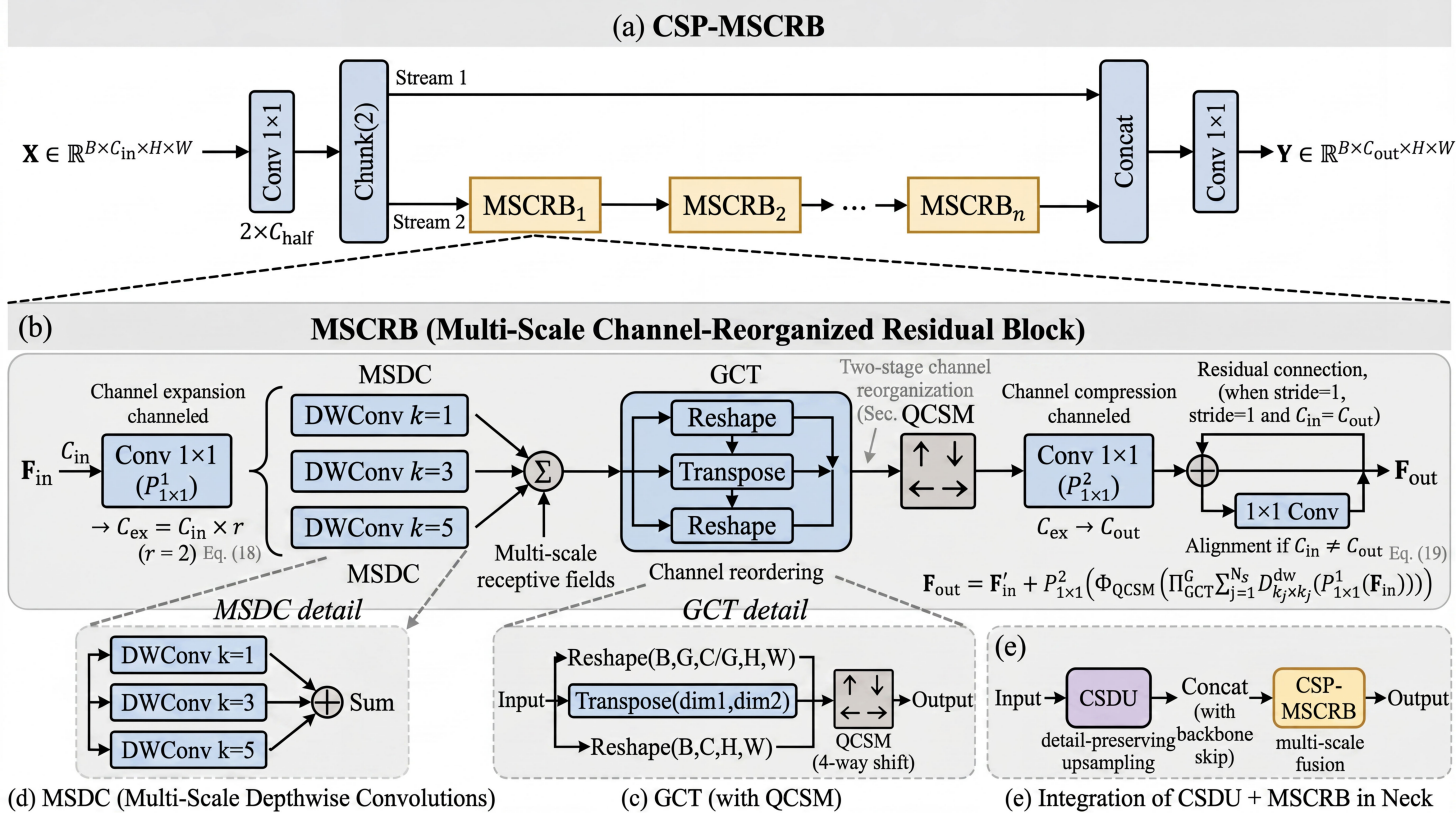
Architecture of CSP-MSCRB. Panel **(a)** shows the top-level CSP topology: the input $X\in {\mathbb{R}}^{B\times C_{\text{in}}\times H\times W}$ is projected and split into two streams; Stream 2 is refined by *n* cascaded MSCRB blocks before being concatenated with the identity Stream 1 and projected to $Y\in {\mathbb{R}}^{B\times C_{\text{out}}\times H\times W}$. Panel **(b)** details one MSCRB block: a 1×1 convolution expands the channel dimension to *C*_ex_=*r* · *C*_in_, default *r* = 2, Equation 19; the expanded feature is fed into three parallel depthwise branches [MSDC, kernel sizes {1, 3, 5}, Panel **(d)**] whose outputs are summed to form a compact multi-scale representation; a two-stage channel reorganization — GCT (Panel **(c)**, a parameter-free reshape-transpose-reshape under *G* groups), followed by QCSM (identical in role to **Figure 4**, four-way cyclic shift) — then improves channel-spatial interaction; finally a 1×1 convolution compresses the feature to *C*_out_ (Equation 20). A residual path with optional channel alignment is added when stride *s* = 1. Panel **(e)** illustrates the co-placement of CSDU (detail-preserving upsampling) and CSP-MSCRB (multi-scale fusion) in the neck.

**Figure 6:**
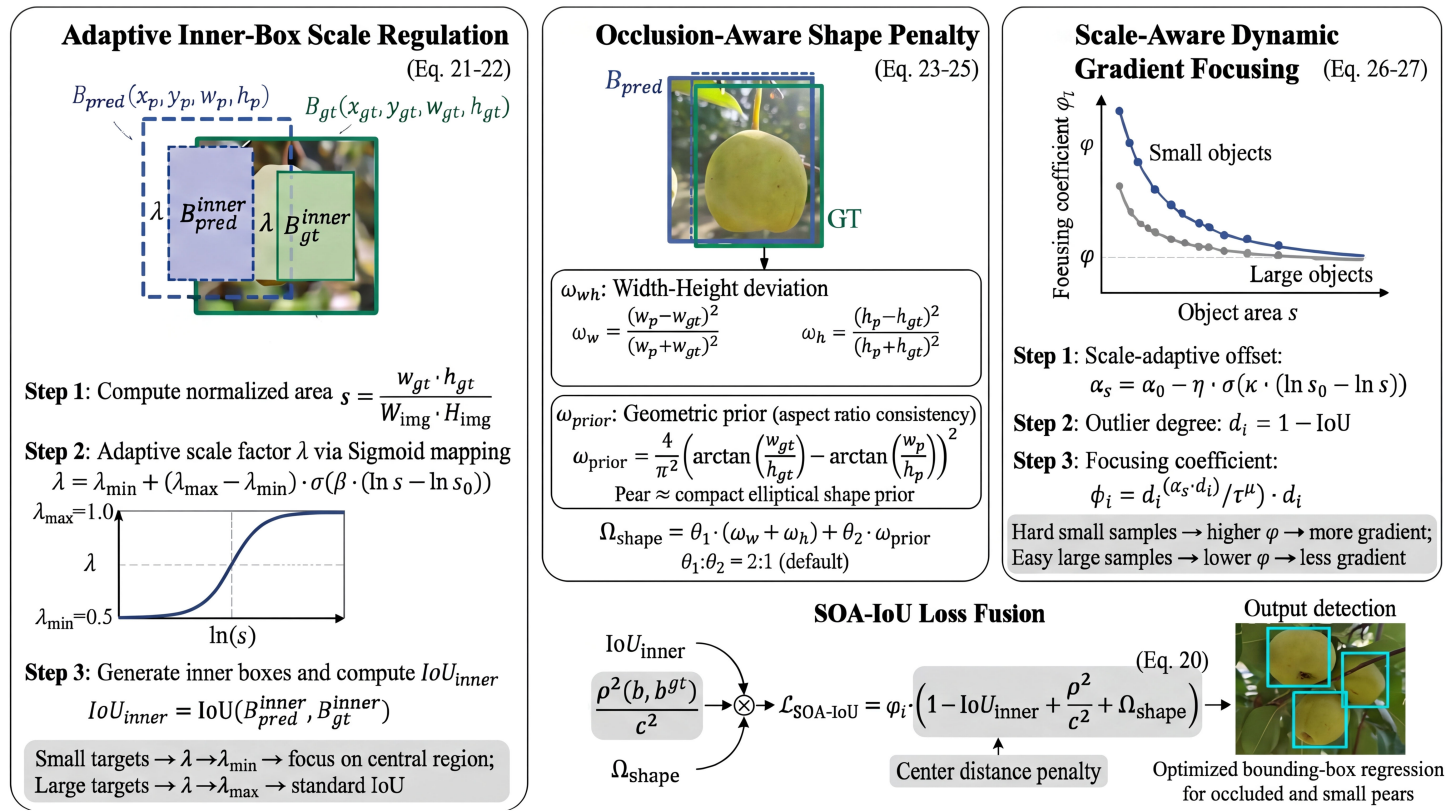
Illustration of SOA-IoU. The three panels correspond to the three coordinated terms in the total loss (Equation 21). The left panel shows adaptive inner-box scale regulation: the normalized target area is mapped through a sigmoid to the adaptive scale factor (Equation 22), which is used to construct inner boxes and compute the inner-box IoU (Equation 23). The middle panel shows the occlusion-aware shape penalty, composed of the width-height deviation and the aspect-ratio prior, combined with the default weight ratio (Equation 26). The right panel shows scale-aware dynamic gradient focusing: the scale-adaptive offset (Equation 27) and the outlier degree jointly define the focusing coefficient (Equation 28), which assigns higher gradient weight to small and hard samples and lower weight to easy large samples. The bottom block summarizes how the three terms are fused. All symbols in the figure match those in Equations 21-28; no additional variables are introduced.

To maintain visual consistency across all architectural illustrations, **Figures 1**–**6** adopt a unified set of drawing conventions. Solid arrows denote forward data flow, dashed arrows denote skip or identity connections, and dotted arrows (where present) indicate training only signals such as noise injection. Module blocks are color-coded by functional category: proposed modules in orange, backbone modules in blue, neck modules in green, and standard operations in gray, as summarized in the legend of **Figure 1**. Dashed borders enclose magnified detail panels, which are linked to their parent blocks by gray leader lines. All tensor shapes are annotated in B × *C* × *H* × *W* format alongside the corresponding data paths.

Specifically, CSP-SGLFE is inserted into the backbone stages to strengthen contextual representation through efficient long-range dependency modeling. SGEAE is applied to pyramid features to replace fixed transformation with input-adaptive sparse enhancement. In the neck, CSDU is used for detail-preserving upsampling, and MSCRB is applied after feature aggregation to improve multi-scale structural representation. During training, the default IoU loss is replaced with SOA-IoU to improve localization for small and partially occluded fruits. The detection head is kept unchanged. The resulting network remains lightweight while improving feature representation, multi-scale fusion, and bounding-box regression.

To provide an algorithmic summary of the complete pipeline, the overall procedure is presented in Algorithm 1.

**Notation.** Throughout Section 2, B denotes the batch size (distinguished from the SSD input-projection matrix *B*_in_ introduced in Section 2.2.2), *C* denotes the channel width at the current stage, and *H*×*W* denotes the spatial resolution. When a feature is reshaped into sequence form, *L*=*H*×*W* is the flattened sequence length. Tensor shapes are denoted by ${\mathbb{R}}^{B\times C\times H\times W}$ for spatial features and ${\mathbb{R}}^{B\times C\times L}$ for sequence features. Element-wise multiplication is denoted by ⊙; concatenation along the channel dimension is denoted by Concat*_C_*(·); the Iverson bracket **1**[·] equals 1 when the condition inside holds and 0 otherwise. The Sigmoid and GELU activations are denoted by *σ*(·) and *σ_G_*(·) respectively.

Algorithm 1

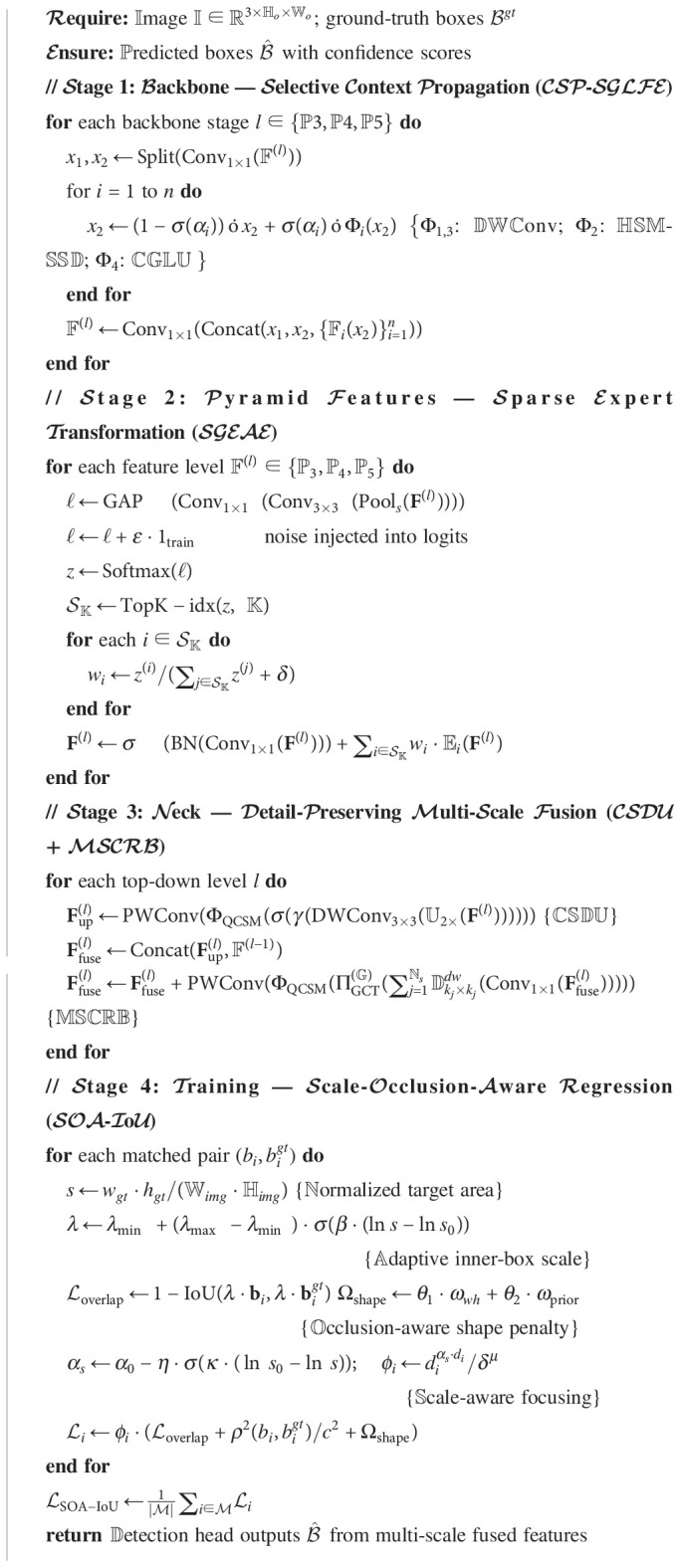

### 2.2 Selective context propagation via the CSP-SGLFE module

To address this trade-off, we develop an efficient gated global–local state-space feature enhancement module, termed CSP-SGLFE. The use of state-space modeling here, rather than self-attention, follows from the deployment budget (Gu and Dao, 2024; Dao and Gu, 2024; Liu et al., 2024b). At the P3 level the flattened sequence length reaches *L* = 6400, where the *O*(*L*^2^) cost of softmax attention becomes impractical for embedded inference, whereas the linear *O*(*C* · *N* · *L*) cost of state-space modeling with *N* = 64 ≪ *L* remains tractable (quantified in Section 2.2.2). Long-range context is thereby made affordable at precisely the high-resolution stages where small and occluded pears most require it. As shown in **Figure 2**, by combining hierarchical state-space modeling with gated local modulation, the module preserves global semantic dependencies relevant to fruit recognition while retaining sensitivity to local textures and boundaries, thereby providing more stable and discriminative features for subsequent transformation and fusion.

#### 2.2.1 Structural design and workflow of the CSP-SGLFE module

The proposed CSP-SGLFE module follows the cross-stage partial topology of C3k2, but substantially redesigns its core transformation pathway to improve selective context propagation under lightweight constraints. Given an input feature map $X\in {\mathbb{R}}^{B\times C_{1}\times H\times W}$, where B denotes the batch size, the module first applies a 1 × 1 pointwise convolution to project the input into 2*c* hidden channels, where *c* = *C*_2_ · *e* and *e* denotes the channel expansion ratio. The projected feature is then evenly split along the channel dimension into two parallel streams, denoted by *x*_1_ and *x*_2_. The branch *x*_1_ serves as an identity-preserving shortcut to retain low-level structural details, whereas *x*_2_ is fed sequentially into *n* cascaded SGLFE blocks for progressive global-local joint modeling. The outputs of these blocks are concatenated together with *x*_1_ along the channel dimension and finally compressed to the target output dimension *C*_2_ through another 1 × 1 pointwise convolution. Let *y*_0_ = *x*_2_ and recursively define *y_i_*= *F_i_*(*y_i_*_−1_), where *F_i_*(·) denotes the transformation of the *i*-th SGLFE block. The overall output is written as

(2) $$Y={\text{Conv}}_{1\times 1}({\text{Concat}}_{C}(x_{1},y_{0},y_{1},\ldots ,y_{n}))$$

where Concat*_C_*(·) denotes concatenation along the channel dimension. The input split is given by Equation 3:

(3) $$x_{1},x_{2}=\text{Split}({\text{Conv}}_{1\times 1}(X))$$

and *F_i_*(·) denotes the nonlinear transformation of the *i*-th SGLFE block. The CSP short gradient path is preserved by the ungated *x*_1_ branch in Equation 2, whose contribution to *Y* does not depend on any *α_l_*. The gated form inside the SGLFE block then controls, per-stage, how aggressively each Φ*_l_*rewrites this feature: *σ*(*α_l_*) → 0 degenerates the *l*-th stage to an identity mapping, while *σ*(*α_l_*) → 1 lets Φ*_l_*dominate. Because ${\left\{\alpha_{l}\right\}}_{l=1}^{4}$ are learned per channel, different channels can adopt different trade-offs between detail preservation and semantic modeling without being constrained by a globally fixed residual weight.

#### 2.2.2 Formulation of the SGLFE block

The SGLFE block is the core computational unit of CSP-SGLFE. It adopts a four-stage cascaded adaptive residual design to integrate local spatial inductive bias, global sequence modeling, local feature refinement, and gated feed-forward enhancement within a unified lightweight structure. To adaptively control the contribution of each stage, the block introduces learnable scaling parameters $\alpha \in {\mathbb{R}}^{4\times C}$, which are constrained to (0, 1) through a Sigmoid activation. Given an input feature $x_{0}\in {\mathbb{R}}^{B\times C\times H\times W}$, the four stages can be uniformly formulated as:

(4) $$x^{\left(l\right)}=(1-\sigma (\alpha_{l}))⊙x^{\left(l-1\right)}+\sigma (\alpha_{l})⊙{\text{Φ}}_{l}(x^{\left(l-1\right)}),\;l=1,2,3,4$$

where *σ*(·) denotes the Sigmoid function, $\alpha_{l}\in {\mathbb{R}}^{C}$ is the learnable scaling vector of the *l*-th stage, and ⊙ denotes element-wise multiplication. Since $\alpha_{l}\in {\mathbb{R}}^{C}$ while $x^{\left(l-1\right)}\in {\mathbb{R}}^{alB\times C\times H\times W}$*^W^*, the gate *σ*(*α_l_*) is broadcast along the batch and spatial dimensions before element-wise multiplication. The four transformation functions Φ*_l_*(·) correspond to different functional submodules. Specifically, Φ_1_ is a 3 × 3 depthwise convolution for capturing local short-range textures and boundary cues; Φ_2_ is the hierarchical state-space sequence modeling module (HSM-SSD), which models long-range dependencies with linear complexity; Φ_3_ is another 3 × 3 depthwise convolution for local spatial smoothing and refinement after global modeling; and Φ_4_ is a convolutional gated linear unit (CGLU), which performs selective channel-wise modulation in the feed-forward stage.

Among the stages, HSM-SSD provides the key mechanism for efficient global context aggregation. To avoid notational ambiguity between the batch dimension and the SSD input projection matrix (conventionally also denoted *B* in the state-space literature), we use B for batch size and rename the SSD input and output projections as *B*_in_ and *C*_out_ respectively throughout this subsection; we follow (Dao and Gu, 2024) for the remaining conventions. Let $\bar{x}\in {\mathbb{R}}^{B\times C\times L}$ denote the flattened feature sequence after channel normalization, where *L* = *H* × *W*. HSM-SSD first applies three pointwise projections to $\bar{x}$ to produce:

- an input stream $u\in {\mathbb{R}}^{B\times C\times L}$ and a gating stream $z\in {\mathbb{R}}^{B\times C\times L}$;
- state-space parameters $B_{\text{in}}\in {\mathbb{R}}^{B\times N\times L}$, $C_{\text{out}}\in {\mathbb{R}}^{B\times N\times L}$ and a discretization step $\text{Δ}\in {\mathbb{R}}^{B\times N\times L}$,

where *N* = 64 is the state dimension.

A 3 × 3 depthwise convolution is applied to *u* to inject local spatial priors before the state-space recurrence. Unlike standard Mamba, which discretizes the state-space parameter through $\bar{A}=\exp\;(\text{Δ}⊙A)$), HSM-SSD replaces the exponential discretization with a hierarchical softmax normalization along the sequence dimension:

(5) $$\begin{array}{ll} \bar{A} & ={\text{Softmax}}_{L}(\text{Δ}+A_{\text{init}})\in {\mathbb{R}}^{B\times N\times L},\\ \text{h} & =\text{SSM}(\bar{A},B_{\text{in}},C_{\text{out}},u)\in {\mathbb{R}}^{B\times C\times L},\\ \hat{y} & =\text{h}⊙\text{SiLU}(z)+u⊙D_{s}\in {\mathbb{R}}^{B\times C\times L} \end{array}$$

Here $A_{\text{init}}\in {\mathbb{R}}^{N}$ is a learnable diagonal initialization parameter, broadcast to ${\mathbb{R}}^{B\times N\times L}$ along the batch and sequence dimensions before being added to Δ; Softmax*_L_*(·) denotes softmax along the token-length dimension *L*; SSM(·) is the standard state-space scan operator implemented as a batched parallel associative scan (Dao and Gu, 2024), in which *B*_in_ and *C*_out_ act as per-token input- and output-projection matrices, and $D_{s}\in {\mathbb{R}}^{C}$ is a learnable per-channel skip-scaling vector that provides a direct pathway from *u* to *y*.

Replacing exp(·) by Softmax(·) is motivated by the observation that, in detection feature maps, the effective influence of each state token is bounded and sparsity-biased rather than geometrically decayed over time. Enforcing $\sum_{t=1}^{L}{\tilde{\text{Δ }}}_{\left\{b,n,t\right\}}=1$ per sample *b* and per state channel *n* stabilizes training when *L* varies across the three FPN levels (*L* = 6400, 1600, and 400 for P3, P4, and P5, respectively), while keeping the linear complexity of SSD unchanged.

This formulation yields linear computational complexity with respect to sequence length. Each HSM-SSD call requires O(B·L·N·D) multiply-accumulate operations, and its attention-like state matrix is never materialized in memory, so the peak activation memory scales as O(B·L·N). For reference, a vanilla softmax self-attention block at the same feature resolution requires $O(B\cdot L^{2}\cdot D)$ operations and $O(B\cdot L^{2})$ memory for the attention matrix.

The asymptotic gain therefore becomes meaningful only when *L* ≫ *N*. In our detection setting this condition is satisfied at the two highest-resolution stages: at the P3 level *L* = 80^2^ = 6400 and at the P4 level *L* = 40^2^ = 1600, both well above *N* = 64, giving a theoretical FLOPs ratio of approximately 100× and 25×, respectively, in favor of HSMSSD. At the P5 level (*L* = 400) the gap narrows, and the benefit of HSM-SSD lies primarily in its bounded memory footprint rather than FLOPs. We emphasize that these figures are asymptotic; actual wall-clock speed-ups depend on memory-access patterns, kernel fusion, and hardware-specific optimizations, and are typically smaller than the theoretical ratio but still practically significant for edge deployment (Dao and Gu, 2024).

In the final stage, CGLU acts as a gated feed-forward module for nonlinear channel enhancement and selective information regulation. Unlike a conventional feed-forward network with fixed channel transformation, CGLU introduces a depthwise-convolution-based gating branch so that channel responses are modulated in a spatially aware manner. Its formulation is:

(6) $$\begin{array}{ll} \left[x_{a},x_{b}]=\text{Split}({\text{Conv}}_{1\times 1}^{W_{1}}(x_{\text{in}})\right)\\ x_{\text{out}} & =x_{\text{in}}+{\text{Conv}}_{1\times 1}^{W_{2}}(\sigma_{G}({\text{DWConv}}_{3\times 3}(x_{a}))⊙x_{b}) \end{array}$$

where ${\text{Conv}}_{1\times 1}^{W_{1}}$ is a pointwise convolution with weight $W_{1}\in {\mathbb{R}}^{2d^{\prime }\times C}$ that expands the channel width from *C* to 2*d*^′^; Split(·) divides the expanded tensor evenly along channels into a transformation branch $x_{a}\in {\mathbb{R}}^{B\times d^{\prime }\times H\times W}$ and a gating branch *x_b_*of the same shape; DWConv_3×3_(·) is a 3 × 3 depthwise convolution; $\sigma_{G}(\cdot )$ is the GELU activation; and ${\text{Conv}}_{1\times 1}^{W_{2}}$ with $W_{2}\in {\mathbb{R}}^{C\times d^{\prime }}$ projects the gated feature back to *C* channels for the residual addition. Through this gated interaction, irrelevant channel responses can be suppressed while informative responses are retained. This mechanism is beneficial in orchard scenes, where fruit-related features are often contaminated by background interference from branches, leaves, and sky regions. The SGLFE block thus integrates local, global, and gated modulation in a residual framework.

### 2.3 Selective transformation path allocation via the SGEAE module

Where CSP-SGLFE selects what contextual information to propagate, SGEAE addresses a complementary question: which transformation pathway should process a given input. It realizes this through an explicit sparse gating mechanism in which only a Top-K subset of candidate transformations is activated per input, with the selection itself conditioned on spatial content. Because orchard degradations vary substantially across scenes, fixed transformation pathways are often inadequate under limited computational budgets. To address this limitation, we propose a sparse gated expert aggregation enhancement module (SGEAE), which dynamically assigns the most relevant transformation routes through a spatially aware routing mechanism. As shown in **Figure 3**, by combining a shared baseline stream with a sparse expert stream for conditional enhancement, SGEAE enables input-adaptive feature transformation while preserving lightweight computation, thereby improving robustness to diverse orchard-specific degradations.

#### 2.3.1 Overall design of SGEAE

SGEAE decouples feature enhancement into an always-on shared stream and a conditionally activated sparse expert stream, fused additively to balance stable delivery with input-adaptive specialization.

The departure from conventional mixture-of-experts routing is deliberate. Token-level gating through fully connected layers discards spatial layout, yet in orchard scenes the occlusion pattern varies across spatial regions: one part of a fruit cluster may be branch occluded while an adjacent part remains visible. SGEAE therefore routes through a convolution-based spatially aware mechanism (SAGR, Section 2.3.2) that preserves this structure, allowing different regions to draw on different experts according to their local visibility. The largest gain of this module falls on the branch-leaf occlusion subset (**Table 1**), consistent with this design.

Given an input feature tensor $X\in {\mathbb{R}}^{B\times C\times H\times W}$, SGEAE first employs a spatial-aware gating router (SAGR) to analyze the global semantic characteristics of the input and determine a sparse subset of experts to be activated according to a Top-*K* selection strategy. In parallel, the shared pathway applies a lightweight 1 × 1 convolutional transformation to provide a stable baseline representation, whereas the selected expert branches perform heterogeneous nonlinear transformations according to their respective structures. Let ${\left\{w_{i}\right\}}_{i=1}^{N}$ denote the unnormalized routing scores produced by SAGR (Section 2.3.2). The conditional output of SGEAE can be written in a gated mixture-of-experts form:

(7) $$Y=f_{\text{shared}}(X)+\overset{N}{\underset{i=1}{\sum }}w_{i}\cdot E_{i}(X)\cdot 1[i\in S_{K}]$$

where *Y* is the enhanced output, *f*_shared_(·) is the shared baseline mapping, *E_i_*(·) is the transformation function of the *i*-th expert, *S_K_*is the set of Top-K expert indices selected by SAGR, and **1**[·] is the Iverson bracket. Experts outside *S_K_*are masked out in both the forward pass and the gradient computation, which is the mechanism that enables conditional computation. In the actual implementation, the raw scores *w_i_*in Equation 7 are replaced by their Top-K-restricted softmax renormalization, giving the concrete form.

Purely sparse routing risks expert collapse during early training, a well-documented failure mode in mixture-of-experts systems where one or two experts dominate the routing decisions and the remaining experts receive too few gradients to specialize (Shazeer et al., 2017). To mitigate this, we adopt a dual-stream architecture (DSSEA) in which the shared stream provides a stable baseline and only the Top-K experts are conditionally activated. The shared stream is implemented as a lightweight 1 × 1 convolution followed by batch normalization and SiLU activation, providing a stable low-frequency semantic basis for all inputs without routing uncertainty. The sparse stream contains N heterogeneous expert subnetworks, among which only the K experts selected by SAGR participate in computation.

With the shared stream in place, the actual computation of SGEAE instantiates Equation 7 by fusing the shared and sparse streams through element-wise addition:

(8) $$Y=\sigma (\text{BN}({\text{Φ}}_{1\times 1}(X)))+\underset{i\in S_{K}}{\sum }\frac{w_{i}}{\sum_{j\in S_{K}}w_{j}+\delta }\cdot E_{i}(X)$$

where *σ*(·) denotes the SiLU activation function and *δ* = 10^−6^ is a small constant for numerical stability. Even if routing decisions are suboptimal in the early stage of training, the shared branch still guarantees a stable baseline representation, which suppresses gradient oscillation and mitigates expert collapse.

Empirical verification. To verify that the dual-stream design achieves the intended balancing effect, we recorded per-expert activation frequencies on the Orchard Pear validation set after training converged, and computed the normalized load-balance coefficient $L_{\text{bal}}=(N/K^{2})\sum_{i=1}^{N}f_{i}p_{i}$, where *f_i_*is the fraction of tokens routed to expert *i* and *p_i_*is its average routing probability. Under perfectly uniform utilization, $L_{\text{bal}}\to 1$; larger values indicate routing imbalance. For the full dual-stream SGEAE (*N* = 4*, K* = 2), the four experts were activated with frequencies {0.27, 0.25, 0.26, 0.22}, yielding $L_{\text{bal}}=1.14$, close to the ideal balance. Removing the shared stream caused the distribution to skew sharply to {0.61, 0.18, 0.13, 0.08}, raising $L_{\text{bal}}$ to 1.89—clear evidence that a single expert dominates routing when the stabilizing shared pathway is absent. The full expert-utilization histogram across training epochs is reported in **Supplementary Appendix Figure 12**.

Sensitivity to *K* and *N*. The default setting *N* = 4, *K* = 2 is the configuration on the accuracy–efficiency Pareto frontier of our deployment target, identified by a grid search over *N* ∈ {2, 4, 6, 8} and *K* ∈ {1*, …, N*}. Two regularities emerged from the sweep: (i) the sparsity ratio *K/N* ≈ 0.5 consistently outperforms both denser activation (*K/N* = 0.75) and stricter sparsity (*K/N* = 0.25) at each value of *N*, because excessive activation incurs redundant computation while overly sparse routing loses transformation diversity; and (ii) increasing *N* beyond 4 yields only marginal accuracy gains (≤ 0.2% mAP@50) but inflates parameters by 13–29%, making *N* = 4 the most deployment-friendly choice. The full sensitivity table is provided in **Supplementary Appendix Table 7**.

#### 2.3.2 Spatial-aware gating router

The spatial-aware gating router (SAGR) is the key decision-making unit in SGEAE for conditional sparse expert activation. Unlike conventional MoE routing mechanisms that rely on fully connected token-level gating, SAGR adopts a convolution-based spatial-aware routing strategy to preserve structural information during expert selection. This design is more suitable for dense prediction tasks such as fruit detection, where spatial layout and local neighborhood patterns are essential for reliable feature transformation.

To reduce routing overhead, the input is first adaptively average-pooled when its spatial resolution exceeds a predefined threshold. The reduced feature is then processed by a two-stage convolutional routing network. The first stage is a 3 × 3 convolution followed by batch normalization and SiLU activation, which compresses the channel dimension from *C* to max(*C/r*, 8) and extracts local spatial texture patterns relevant to routing. The second stage is a 1 × 1 pointwise convolution that maps the compressed feature to *N* channels, where each channel corresponds to the preference score of one expert at each spatial location. After global average pooling, a batch-level expert selection logits vector is obtained.

During training, Gaussian noise $\epsilon ∼N(0,\sigma_{\epsilon }^{2}I)$ with *σ_ϵ_*= 1.0 is added to the routing logits GAP(*z*_3_) before the Softmax normalization, so that exploration is injected at the logit level and the resulting routing probabilities remain a valid distribution over the simplex (Shazeer et al., 2017). The Top-*K* experts are then selected by ranking the routing probabilities, and their scores are locally renormalized to produce the mixing weights used in Equation 8. The overall routing process is given by Equations 9–13, in which the pooled input (Equation 9) is encoded by a 3×3 convolution (Equation 10) and a 1×1 convolution (Equation 11), normalized by Softmax (Equation 12), and used for Top-K expert selection and weight renormalization (Equation 13):

(9) $$z_{1}={\text{Pool}}_{s}(X)$$

(10) $$z_{2}=\sigma (\text{BN}({\text{Φ}}_{3\times 3}(z_{1})))$$

(11) $$z_{3}=\text{BN}({\text{Φ}}_{1\times 1}(z_{2}))$$

(12) $$z_{4}=\text{Softmax}(\text{GAP}(z_{3})+\epsilon \cdot 1_{\text{train}})$$

(13) $$S_{K}=\text{TopK}-\text{idx}(z_{4},K),\;w_{i}=\frac{z_{4}^{\left(i\right)}}{\sum_{j\in S_{K}}z_{4}^{\left(j\right)}+\delta }\text{ for }i\in S_{K}$$

where Pool*_s_*(·) denotes adaptive average pooling with spatial downsampling factor *s*, Φ_3×3_ and Φ_1×1_ are the 3 × 3 channel-reduction convolution and the 1 × 1 expert-mapping convolution respectively, BN(·) denotes batch normalization, GAP(·) denotes global average pooling over the spatial dimensions, **1**_train_ is the training-mode indicator (1 during training, 0 at inference), TopK-idx(·*, K*) returns the indices of the *K* largest entries, and *δ* = 10^−6^ is a small constant shared with Equation 8 for numerical stability.

Compared with fully connected routing, SAGR preserves spatial cues before global aggregation. At the same time, the combination of spatial downsampling and channel compression ensures that the routing process itself remains computationally lightweight and does not become a bottleneck for embedded deployment. Through this design, SAGR enables SGEAE to dynamically activate the most relevant transformation paths for inputs affected by occlusion, scale variation, and complex background interference, which improves feature adaptability and transformation efficiency in unstructured orchard scenes.

### 2.4 Selective detail reorganization in lightweight multi-scale fusion

The preceding modules enhance individual feature maps; the remaining challenge is how to preserve their discriminative content during multi-scale fusion. CSDU and MSCRB address this at the fusion level: rather than routing features through input-dependent gates, they allocate the limited fusion capacity toward preserving fine structural details—through parameter-free directional channel mixing and multi-scale depthwise branching—that are most vulnerable to loss in conventional top-down interpolation. Top-down feature transmission in lightweight necks often suffers from interpolation smoothing and insufficient channel interaction, which weakens local contrast and leads to missed detections of small fruits. To address this problem, we design a collaborative fusion strategy based on the channel-shuffle-enhanced depthwise upsampling module (CSDU) and the multi-scale channel-reorganized residual block (MSCRB). The distinction from standard channel shuffle is central to this design. Conventional shuffle reorders channel indices but leaves spatial content untouched, whereas recovering fine detail during upsampling, on which dense fruit separation and small-target recognition depend, requires reintroducing local spatial cues. As shown in **Figure 4**, the quadrant mixer (QCSM) within CSDU adds directional cyclic shifts on top of channel grouping, so that each channel group perceives a neighboring spatial position at no parameter cost, while MSCRB complements this with multi-branch receptive fields and channel-reorganized residual learning across scales. Together the two modules protect the local structures most readily eroded in conventional top-down interpolation.

#### 2.4.1 Structure of CSDU

The proposed CSDU module decomposes the upsampling process into four cascaded stages: spatial resolution recovery, per-channel spatial refinement, cross-channel spatial interaction, and channel-wise linear projection. This design aims to improve detail-preserving feature reconstruction while retaining low computational cost.

Given an input feature map $F_{\text{in}}\in {\mathbb{R}}^{B\times C\times H\times W}$, CSDU first enlarges the spatial resolution by a factor of 2 using nearest-neighbor interpolation. The upsampled feature is then refined by a 3 × 3 depthwise convolution with the number of groups equal to the number of channels *C*, so that each channel is processed independently in the spatial domain. Batch normalization and SiLU activation are subsequently applied to complete normalization and nonlinear mapping. Although depthwise convolution is highly efficient, its channel-wise independence leads to insufficient information exchange across channels. To address this limitation, the third stage introduces the quadrant channel-spatial mixer (QCSM), which splits the feature into four channel groups and applies directional cyclic spatial shifts to establish spatial interaction across channels without introducing learnable parameters. Finally, the mixed feature is linearly projected by a 1 × 1 pointwise convolution to obtain the output feature.

The overall transformation is formulated as Equation 14:

(14) $$F_{\text{out}}=P_{1\times 1}({\text{Φ}}_{\text{QCSM}}(\sigma (\gamma (D_{k\times k}^{\text{dw}}(U_{2\times }(F_{\text{in}}))))))$$

where *U*_2×_(·) denotes 2× nearest-neighbor upsampling, $D_{k\times k}^{\text{dw}}(\cdot )$ denotes depthwise convolution with kernel size *k* × *k* and group number *C*, γ(·) denotes batch normalization, *σ*(·) denotes the SiLU activation, ${\text{Φ}}_{\text{QCSM}}(\cdot )$ denotes the quadrant channel-spatial mixing operation, and *P*_1×1_(·) denotes pointwise convolution. Since QCSM is parameter-free, CSDU remains lightweight while improving cross-channel interaction during upsampling.

#### 2.4.2 Quadrant channel-spatial mixer

QCSM is the core component of CSDU for alleviating the channel isolation problem introduced by depthwise convolution. Conventional channel shuffle mainly reorders channel indices and does not explicitly involve spatial information exchange. In contrast, QCSM introduces directional cyclic shifts on top of channel grouping, allowing different channel groups to perceive neighboring spatial locations and to establish cross-channel spatial interaction in a parameter-free manner.

Given an input feature map $X\in {\mathbb{R}}^{B\times C\times H\times W}$, QCSM first evenly splits it into four channel groups along the channel dimension:

(15) $$\left\{X^{\left(1\right)},X^{\left(2\right)},X^{\left(3\right)},X^{\left(4\right)}\}=S_{C}^{4}(X\right)$$

where $S_{C}^{4}(\cdot )$ denotes four-way channel partition operator. Each sub-feature *x*^(^*^i^*^)^ is then subjected to a directional cyclic shift along either the height or the width dimension:

(16) $${\tilde{X}}^{\left(i\right)}=T_{d_{i},\delta_{i}}(X^{\left(i\right)}),\;i\in \{1,2,3,4\}$$

where *T_di_, δ_i_* denotes the cyclic shift operator with step size *δ* along spatial dimension *d*, implemented as a zero-parameter tensor roll.

(17) $$\left\{(d_{1},\delta_{1}),(d_{2},\delta_{2}),(d_{3},\delta_{3}),(d_{4},\delta_{4})\}=\{(H,+s),(H,-s),(W,+s),(W,-s)\right\}$$

with shift step *s* = 1 in our implementation. These four (direction, step) pairs exactly correspond to the four colored boxes in the QCSM sub-panel of **Figure 4**, whose internal arrows visualize the up, down, left, and right cyclic shift directions, respectively. The four shifted sub-features are then concatenated along the channel dimension to recover the original feature shape:

(18) $${\text{Φ}}_{\text{QCSM}}(X)={\text{Concat}}_{C}({\tilde{X}}^{\left(1\right)},{\tilde{X}}^{\left(2\right)},{\tilde{X}}^{\left(3\right)},{\tilde{X}}^{\left(4\right)})$$

Through this design, the four channel groups encode neighboring information from the up, down, left, and right directions, respectively. In this way, QCSM effectively enlarges the equivalent receptive field of each channel and increases the spatial perception diversity of subfeature groups without introducing extra parameters. Compared with standard channel shuffle, QCSM not only reorganizes channel order but also explicitly injects local spatial displacement cues, which is particularly beneficial for recovering fine structural details during upsampling. This mechanism is well suited to real-time detection scenarios, where stronger channel-spatial interaction is required under strict efficiency constraints.

#### 2.4.3 Multi-scale channel-reorganized residual block

To further enhance multi-scale feature extraction and complement CSDU in the fusion stage, we propose the multi-scale channel-reorganized residual block (MSCRB), as illustrated in **Figure 5**. For clarity, we use MSCRB to denote a single block and CSPMSCRB to denote the complete neck-fusion module—*n* MSCRB blocks wrapped in a cross-stage-partial topology (**Figure 5a**); the two names are used consistently in this sense throughout. The module is designed to improve scale-aware structural representation through parallel lightweight receptive fields and hierarchical channel reorganization, while maintaining efficient inference. Given an input feature $F_{\text{in}}\in {\mathbb{R}}^{B\times C_{\text{in}}\times H\times W}$, MSCRB first expands the channel dimension to $C_{\text{ex}}=r\cdot C_{\text{in}}$ via a 1×1 pointwise convolution $P_{1\times 1}^{\left(1\right)}(\;\cdot \;)$, where *r* is the channel expansion ratio (default *r* = 2). The expanded feature is then fed into three parallel depthwise branches with kernel sizes *k_j_*∈ {1, 3, 5}, so as to capture spatial information under different receptive fields. Rather than concatenating the branch outputs, which would inflate the channel dimension and computational cost, the three outputs are aggregated by element-wise summation to form a compact multi-scale representation:

(19) $$F_{\text{ms}}=\overset{3}{\underset{j=1}{\sum }}{\text{D}}_{k_{j}\times k_{j}}^{\text{dw}}(P_{1\times 1}^{\left(1\right)}(F_{in})).$$

After multi-scale aggregation, the feature is sequentially processed by grouped channel transposition (GCT) and QCSM, forming a two-stage channel reorganization mechanism. GCT first breaks the fixed correspondence between channel groups, and QCSM subsequently injects directional spatial interaction across the reorganized channels. This combination enhances both channel diversity and local structural perception. Finally, a 1 × 1 pointwise convolution compresses the channels back to the target output dimension *C*_out_, resulting in an hourglass-like expand-transform-compress pipeline. When the stride is 1, a residual connection is introduced for identity mapping fusion. If the input and output channel dimensions are inconsistent, an additional 1×1 convolution is used on the residual branch for channel alignment. When the stride is 2, the residual connection is omitted due to the change in spatial resolution. The overall formulation is given by

(20) $$F_{\text{out}}=\{\begin{array}{ll} {F^{\prime }}_{\text{in}}+P_{1\times 1}^{\left(2\right)}({\text{Φ}}_{\text{QCSM}}({\text{Π}}_{\text{GCT}}^{\left(G\right)}(F_{ms}))), & s=1\\ P_{1\times 1}^{\left(2\right)}({\text{Φ}}_{\text{QCSM}}({\text{Π}}_{\text{GCT}}^{\left(G\right)}(F_{ms}))), & s=2 \end{array}$$

where ${F^{\prime }}_{\text{in}}=F_{\text{in}}$ when $C_{\text{in}}=C_{\text{out}}$ and ${F^{\prime }}_{\text{in}}=W_{1\times 1}^{\text{res}}(F_{\text{in}})$ otherwise; $P_{1\times 1}^{\left(2\right)}(\cdot )$ is the pointwise projection that compresses the channel width from $C_{\text{ex}}$ back to $C_{\text{out}}$; ${\text{Π}}_{\text{GCT}}^{\left(G\right)}(\cdot )$ denotes the parameter-free grouped channel transposition with *G* groups; and $F_{ms}$is the multi-scale aggregated feature from Equation 19. When *s* = 2 the spatial resolution changes and the residual connection is omitted, as noted above. By introducing multi-branch depthwise convolution, MSCRB captures spatial semantics at different scales within a single forward pass, which is beneficial for fruits with diverse sizes and partial visibility. The subsequent GCT-QCSM reorganization further improves channel interaction and local structural diversity at negligible overhead. Meanwhile, the residual pathway facilitates gradient propagation and stabilizes optimization in deeper fusion layers. MSCRB and CSDU are complementary: the former strengthens multi-scale representation while the latter improves upsampling detail preservation.

### 2.5 Selective optimization focusing with SOA-IoU

Even with improved features from the preceding three stages, small and partially occluded fruits tend to produce weak regression signals that are easily overwhelmed by the large number of easy samples during training. Conventional IoU-based losses, although effective in general object detection, are not tailored to the scale variation, occlusion, and shape priors of orchard environments. We therefore propose a scale-occlusion-aware adaptive IoU loss, termed SOA-IoU, that reshapes per-sample gradient contributions according to target scale and occlusion degree. The loss combines three terms, each addressing a distinct difficulty: an adaptive inner-box scale stabilizes the IoU of small fruits, whose overlap is otherwise highly sensitive to minor coordinate shifts; a shape regularizer repurposes the aspect-ratio prior so that it continues to constrain partially occluded fruits even when their IoU is already high; and a scale-aware focusing coefficient redirects gradient toward small and occluded hard samples. Rather than acting in isolation, these terms are constructed to operate on largely separate box parameters, and their interaction is analyzed in Section 2.5.4. As shown in **Figure 6**, the combined objective directs training toward the localization challenges most critical for orchard perception, consistent with the representation, transformation, and fusion strategies above.

As illustrated in **Figure 6**, SOA-IoU addresses box regression from three coordinated aspects: adaptive inner-box scale regulation, occlusion-aware shape regularization, and scale-aware dynamic gradient focusing. The overall loss is defined as:

(21) $$L_{\text{SOA}-\text{IoU}}=\phi_{i}\cdot (1-{\text{IoU}}_{\text{inner}}+\frac{\rho^{2}(b,b^{gt})}{c^{2}}+{\text{Ω}}_{\text{shape}})$$

where *b* and *b^gt^* denote the predicted box and ground-truth box, respectively; *ρ*(·) denotes the Euclidean distance between their center points; *c* is the diagonal length of the smallest enclosing box; IoU_inner_ is the IoU computed on adaptively constructed inner boxes; Ω_shape_ is the shape regularization term; and *ϕ_i_*is the scale-aware dynamic focusing coefficient for sample *i*.

#### 2.5.1 Adaptive inner-box scale regulation

For small fruits, even slight localization deviations may cause large fluctuations in IoU, leading to unstable regression optimization. To alleviate this problem, we introduce an adaptive inner-box mechanism that dynamically adjusts the auxiliary box scale according to the normalized target area. Let *s* denote the normalized area of the ground-truth box. The scaling factor *λ* is defined as

(22) $$\lambda =\lambda_{\min\;\;}+(\lambda_{\max\;\;}-\lambda_{\min\;\;})\cdot \sigma (\beta \cdot (\ln\;s-\ln\;s_{0}))$$

where *λ*_min_ and *λ*_max_ denote the lower and upper bounds of the scaling factor, *s*_0_ is the reference area, *β* controls the transition steepness, and *σ*(·) denotes the Sigmoid function. When *s* ≪ *s*_0_, the target is small and *λ* approaches *λ*_min_, so the auxiliary box shrinks more aggressively and the overlap computation focuses on the more reliable central region of the fruit.

When the target becomes larger, *λ* gradually approaches *λ*_max_, and the loss degenerates toward standard IoU behavior. Based on *λ*, the predicted box and the ground-truth box are both scaled to generate their corresponding inner boxes, and the overlap term is computed as

(23) $${\text{IoU}}_{\text{inner}}=\text{IoU}(b^{\text{inner}},b^{gt,\text{inner}})$$

This design reduces the excessive sensitivity of IoU to marginal coordinate perturbations on small objects and thus stabilizes regression learning in dense small-target scenes.

#### 2.5.2 Occlusion-aware shape regularization

In orchard scenes, heavy overlap and branch-leaf occlusion often cause visible fruit regions to become incomplete, which makes bounding-box regression prone to shape distortion. Considering that pears generally exhibit a compact and approximately round geometric structure in images, we introduce an occlusion-aware shape regularization term that jointly constrains size consistency and geometric plausibility.

Let *w_p_*, *h_p_*and *w_gt_*, *h_gt_*denote the width and height of the predicted box and the ground-truth box, respectively. The size consistency termdefined in Equation 24, penalizes width and height mismatches in a scale-invariant manner:

(24) $$\omega_{wh}=\omega_{w}+\omega_{h}=\frac{{\left(w_{p}-w_{gt}\right)}^{2}}{{\left(w_{p}+w_{gt}\right)}^{2}}+\frac{{\left(h_{p}-h_{gt}\right)}^{2}}{{\left(h_{p}+h_{gt}\right)}^{2}}$$

Since pears approximately follow a compact near-elliptical shape prior, we adopt the aspect-ratio consistency term originally proposed as *v* in CIoU (Zheng et al., 2020), and repurpose it here as an occlusion-aware shape regularizer. In CIoU, this term serves as a balance coefficient for the IoU loss; in SOA-IoU it plays a different role: it is explicitly weighted alongside *ω_wh_*in the shape penalty Ω_shape_ (Equation 26), so that it continues to constrain the predicted aspect ratio even when the IoU is already high—which is the typical regime of partially occluded fruits whose visible portion still yields a reasonable IoU but a distorted aspect ratio.

Formally, this aspect-ratio prior term is given by Equation 25:

(25) $$\omega_{prior}=(\frac{4}{\pi^{2}})\cdot {\left[\arctan\;(\frac{w_{gt}}{h_{gt}})-\arctan\;(\frac{w_{p}}{h_{p}})\right]}^{2}$$

The full shape penalty combines the two terms as:

(26) $${\text{Ω}}_{\text{shape}}=\theta_{1}\cdot \omega_{wh}+\theta_{2}\cdot \omega_{prior}$$

where *θ*_1_ and *θ*_2_ are balancing coefficients, set to *θ*_1_:*θ*_2_ = 2:1 by default. The first term enforces explicit size alignment between the predicted and ground-truth boxes, while the second term injects a soft geometric prior that prevents unrealistic aspect-ratio deviations under partial occlusion. By combining explicit size consistency with prior guided regularization, SOA-IoU encourages the detector to recover stable and semantically plausible bounding boxes even when only a limited visible portion of the fruit is available.

#### 2.5.3 Scale-aware dynamic gradient focusing

Another challenge in orchard detection is that hard samples, especially small and occluded fruits, contribute insufficient effective gradients during training because they are dominated by easy and large instances. To address this issue, we design a scale-aware dynamic focusing coefficient that allocates more optimization emphasis to hard regression samples while suppressing harmful gradients from outliers. Let *d_i_*denote the outlier degree of sample *i*, and let the scale-adaptive offset factor be defined as

(27) $$\alpha_{s}=\alpha_{0}-\eta \cdot \sigma (\kappa (\ln\;s_{0}-\ln\;s))$$

where *α*_0_ is the base focusing factor, *s*_0_ is the reference target area shared with Equation 22, *η >* 0 is the maximum scale-adaptive adjustment amplitude, and *κ >* 0 controls the transition steepness around *s*=*s*_0_. With this parameterization, *α_s_*varies continuously over [*α*_0_−*η, α*_0_]: as *s* → 0 (very small targets) the sigmoid approaches 1 and *α_s_*→ *α*_0_−*η*, whereas as *s* → +∞ (very large targets) the sigmoid approaches 0 and *α_s_*→ *α*_0_. Centering the transition at ln*s*_0_ rather than at ln*s* = 0 makes the adjustment symmetric around the typical target size observed in training, which is the quantity actually controlled in Equation 22. Based on *d_i_*and *α_s_*, the focusing coefficient is computed as

(28) $$\phi_{i}=\frac{d_{i}}{e^{\alpha_{s}\cdot d_{i}}\cdot \tau^{\mu }}$$

where *d_i_*∈ [0, 1] is the outlier degree of sample *i*, taken as *d_i_* = 1 − IoU_inner_; *τ* is an exponential moving average of *d_i_*, updated per iteration with momentum 0.9 (initialized to 1), which serves as a batch-level normalizer so that *d_i_/τ* represents a sample’s difficulty relative to the current training regime; and *µ >* 0 is a shaping exponent that controls how sharply *ϕ_i_*concentrates gradient weight on hard samples. The choice of an exponentially normalized *τ* follows the non-monotonic focal mechanism of Wise-IoU v3 (Tong et al., 2023), and prevents the absolute scale of *ϕ_i_*from drifting as the average regression quality improves during training—without this normalization, the effective learning rate of the regression head would decay uncontrollably in later epochs. We set *µ* = 3 in all experiments; the sensitivity to *µ* is reported in **Supplementary Appendix Table 11**. The coefficient *ϕ_i_*is maximized at *d_i_* = 1*/α_s_*; small targets (for which *α_s_*is reduced to *α*_0_−*η* by Equation 27) thus receive their peak gradient weight at a larger outlier degree, shifting optimization effort toward hard small-scale samples. For perfectly matched samples (*d_i_* = 0), *ϕ_i_* = 0 and the sample contributes no gradient, which is the intended focal behavior. Following standard practice for focal-style objectives (Lin et al., 2017), *ϕ_i_*is detached from the computation graph in back-propagation, so it acts purely as a sample-dependent weighting scalar. As a result, the regression objective becomes better aligned with the true error distribution in orchard scenes, where hard examples are sparse but highly influential for final detection quality.

Together, the three terms stabilize small-target overlap, constrain occluded shape, and redirect gradients to hard samples.

#### 2.5.4 Gradient interaction analysis among loss components

Because SOA-IoU combines three distinct mechanisms—adaptive inner-box scaling, occlusion-aware shape penalty, and scale-aware focusing—a natural concern is whether their gradients interfere destructively, particularly under heavy occlusion where the three components are most active simultaneously. We therefore analyze the gradient interaction both analytically and empirically.

Gradient decomposition. Writing the full loss as $L_{\text{SOA}-\text{IoU}}=\phi_{i}\cdot (L_{\text{overlap}}+{\text{Ω}}_{\text{shape}})$, where *ϕ_i_*is the per-sample focusing coefficient, $L_{\text{overlap}}$ is the adaptive inner-box IoU term, and Ω_shape_ is the occlusion-aware shape penalty, the parameter-space gradient takes the form given in Equation 29:

(29) $$\nabla_{\theta }L_{\text{SOA}-\text{IoU}}=\phi_{i}\cdot \nabla_{\theta }L_{\text{overlap}}+\phi_{i}\cdot \nabla_{\theta }{\text{Ω}}_{\text{shape}}+(\nabla_{\theta }\phi_{i})\cdot (L_{\text{overlap}}+{\text{Ω}}_{\text{shape}}))$$

Following the standard design used in focal-style objectives (Lin et al., 2017), we detach *ϕ_i_*from the computation graph during back-propagation, so that ∇*θϕ_i_*= 0 and the third term vanishes. The scale-aware focusing coefficient therefore acts purely as a sample-dependent weighting scalar and cannot itself introduce a new gradient direction. The remaining question is the relationship between $\nabla_{\theta }L_{\text{overlap}}$ and $\nabla_{\theta }L_{\text{shape}}$, which we analyze next.

Parameter-space separation. The two remaining terms operate primarily on different box parameters. The inner-box IoU term is dominated by center-coordinate gradients (*∂/∂x*, *∂/∂y*), since it penalizes misalignment of the central high-confidence region of the prediction with the ground-truth center. The shape penalty, in contrast, is dominated by aspect-ratio gradients (*∂/∂w*, *∂/∂h*), since it penalizes deviation of the predicted width-height ratio from the ground-truth ratio. This separation in the parameter subspace on which the two terms act naturally limits the room for destructive interference.

Behavior under heavy occlusion. Heavy occlusion is precisely the regime where the shape penalty and inner-box scaling could in principle pull in different directions, which is the reason for the occlusion-aware weighting inside Ω_shape_. When a target is detected as heavily occluded (estimated from the intersection ratio between the prediction and the visible portion of the ground truth), the effective weight of the shape penalty is attenuated, so that under severe occlusion the gradient is increasingly dominated by $L_{\text{overlap}}$ and the inner-box center-alignment signal. This design choice was deliberate: when only a fraction of the fruit is visible, forcing the predicted box to match the *full* ground-truth aspect ratio can pull the regression away from the visible evidence. Allowing the shape constraint to relax in this regime preserves the stability of the overlap-driven center localization.

Empirical verification. To verify that the remaining gradient interaction is benign, we sampled a batch of 120 training images from the Orchard Pear validation set, computed $\nabla_{\theta }L_{\text{overlap}}$ and ∇*_θ_*Ω_shape_ with respect to the regression-head parameters for each image, and recorded the cosine similarity between them. Across the sampled batch, the mean cosine similarity is 0.61, indicating moderate positive alignment—the two gradient directions are not redundant (which would correspond to values near 1.0) and are not antagonistic (which would correspond to negative values), but rather cooperate in guiding regression toward the ground-truth box. Stratifying the samples by occlusion severity, the average similarity for heavily occluded fruits (visible area *<* 50%) drops to 0.42, consistent with the intended role of the occlusion-aware weighting in loosening shape enforcement when visual evidence is partial. No samples in the batch exhibit strongly negative cosine similarity (defined as *<* −0.3), confirming the absence of systematic gradient conflict. Taken together, the analytical decomposition, the parameter-space separation, the occlusion-aware relaxation, and the empirical gradient similarity analysis indicate that the three components of SOAIoU operate cooperatively rather than competitively across the full range of orchard scenes encountered during training.

## 3 Results

### 3.1 Datasets

To comprehensively evaluate the proposed framework under both in-domain and cross-domain settings, we conducted experiments on one self-constructed dataset (Orchard Pear) and two publicly available benchmarks (Minne Apple and Mango). The three datasets differ substantially in fruit morphology, density, color distribution, and illumination characteristics, which enables us to assess the generalization ability of the proposed method across diverse orchard scenarios.

#### 3.1.1 Orchard Pear dataset

To evaluate pear detection under realistic field conditions, we constructed a dedicated Orchard Pear dataset for unstructured orchard scenes.

Acquisition and temporal coverage. Image acquisition was conducted in five commercial Korla fragrant pear (*Pyrus sinkiangensis* Yü) orchards in Korla, Xinjiang, China (approximately 41.7°N, 86.1°E), across two consecutive growing seasons: July–September 2023 and July–September 2024. Within each season, acquisition spans three phenological stages of particular relevance for robotic harvesting: late cell division (early-to-mid July), rapid fruit enlargement (late July through August), and color-break to commercial ripeness (early-to-mid September). Images were balanced across these stages, with approximately 30–35% of the dataset captured in each. To capture illumination diversity within a day, acquisitions were distributed over three time windows—direct morning sunlight (08:00– 11:00), diffuse midday conditions (12:00–15:00), and backlit afternoon (16:00–18:00). All images were captured with two handheld cameras (Canon EOS 80D and iPhone 16 Pro Max) at 1920×1080 (video) and 3840×2160 (video) resolutions, respectively, and subsequently resized to 640×640 for training and evaluation.

Geographic and varietal scope. The dataset covers a single geographic region (Korla) and a single cultivar (*Pyrus sinkiangensis* Yü), which bounds its external validity. Regional variation in canopy training systems (e.g., high-spindle vs. Y-trellis), foliage density driven by soil and climate, and cultivar-specific fruit morphology (the elongated Dangshan pear versus the more rounded Korla) would all affect absolute detection performance. To probe how far the pear-specific modules transfer beyond these acquisition conditions, we additionally evaluate cross-scene generalization on two independent public datasets collected under very different conditions: Minne Apple (Häni et al., 2020) and Mango (Koirala et al., 2019). The consistent gains reported on these datasets in Section 3.4.1 provide indirect evidence that the framework captures fruit-detection properties transferable beyond the training conditions.

Dataset composition and splits. The dataset contains 2, 500 images, randomly divided into training, validation, and test sets with a 7:1.5:1.5 ratio. To avoid data leakage, images captured from the same physical shooting scene (defined as the same tree within a 15-minute acquisition window) were constrained to remain in the same subset.

Annotation and inter-annotator analysis. All images were annotated independently by two experienced annotators, and inconsistent annotations were adjudicated by a senior annotator. The inter-annotator agreement, measured as the mean IoU over all matched box pairs, reached 0.92. To characterize the structure underlying this aggregate IoU, we analyzed a randomly sampled subset of 100 disputed boxes, categorizing each disagreement by type. The disagreements were predominantly systematic rather than random in nature: approximately 62% concerned boundary placement on heavily occluded or shadowed fruits (where less than 30% of the fruit surface was visible), 24% concerned visibility threshold decisions for very small instances near the image border (fewer than 10×10 pixels) or partially out-of-frame fruits, 7% concerned instance splitting in tightly clustered fruit groups, and the remaining 7% reflected random labeling noise such as oversight errors. This structure indicates that the residual annotation uncertainty largely reflects genuine visual ambiguity under orchard conditions rather than annotator inconsistency; all such cases were reviewed and resolved by the senior annotator before inclusion in the final dataset.

**Figure 7:**
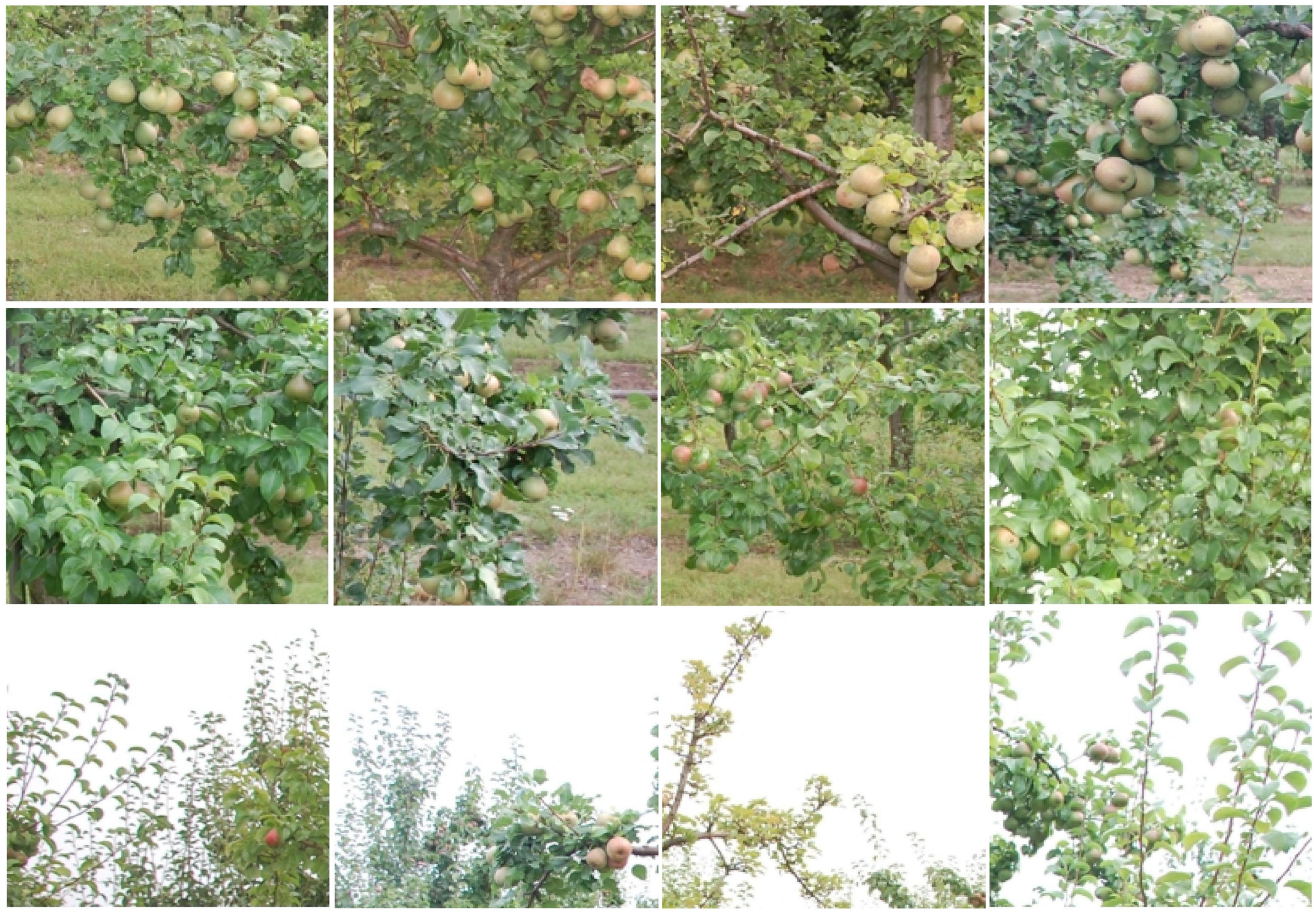
Representative samples from the self-built Orchard Pear dataset, illustrating the three categories of visual degradation that motivate the proposed design. Top row: severe fruit clustering and mutual overlap, which create ambiguous instance boundaries within tight spur clusters even in the absence of branch interference. Middle row: multi-layer branch–leaf and trunk occlusion, which reduces the visible fruit area and weakens local appearance cues. Bottom row: strong direct illumination and backlighting, which introduce large brightness gradients, local overexposure, and color shifts. All images were acquired in five commercial Korla fragrant pear (*Pyrus sinkiangensis* Yü) orchards over two growing seasons (2023–2024) and resized to 640×640 for training and evaluation. The low yellow– green chromatic contrast between mature pears and the surrounding foliage is visible across all rows.

Representative scenes. As shown in **Figure 7**, the dataset systematically covers three representative types of visual degradation in unstructured orchards: (a) severe fruit clustering and overlap, which cause ambiguous boundaries and instance confusion; (b) multi-layer branch–leaf and trunk occlusion, which reduces target visibility; and (c) strong direct illumination and backlighting, which introduce large brightness gradients and color shifts. These characteristics make the Orchard Pear dataset suitable for evaluating the robustness of lightweight detectors in complex orchard environments.

#### 3.1.2 Minne Apple dataset

The Minne Apple dataset (Häni et al., 2020) is a widely used benchmark for apple detection and segmentation in agricultural vision. It was collected in real apple orchards at the University of Minnesota and contains 1, 000 RGB images with 41, 325 annotated apple instances. The dataset spans different fruit maturity stages, with substantial variation in fruit color from green to deep red, as well as pronounced scale variation across instances. Minne Apple was included specifically because its dense small-scale instances reproduce the small-object density that pears exhibit under clustered growth, while its fruit color, ranging from green to deep red, differs markedly from that of pears. This contrast allows the model’s handling of small-scale density to be separated from any reliance on a particular color distribution.

Two characteristics make Minne Apple particularly challenging. First, it is highly dense, with an average of approximately 41 annotated instances per image, which is substantially higher than that of the Orchard Pear dataset. Second, due to the compact canopy structure of apple trees, occlusion and overlap are highly prevalent. These properties make Minne Apple a suitable benchmark for evaluating dense small-object detection. In this study, we follow the original training and test split provided by the dataset authors.

#### 3.1.3 Mango dataset

The Mango dataset (Koirala et al., 2019) for mango detection and yield estimation, contains 1, 730 field images collected in commercial mango orchards in Australia, with approximately 29, 000 annotated mango instances. Compared with the other two datasets, Mango contains relatively larger fruits with a higher single-instance area ratio, while still exhibiting substantial branch–leaf occlusion and illumination variation.

A further challenge of this dataset is its low foreground–background contrast. Mangoes range in color from dark green to yellow-green, often showing limited chromatic difference from surrounding leaves, which increases the difficulty of target–background separation. Mango was included for the complementary reason: this low contrast reproduces the chromatic camouflage that is among the most difficult aspects of pear detection, while its larger fruit scale allows the design to be tested for dependence on a particular target size. Training and evaluation follow the original data split protocol.

### 3.2 Experimental setup

#### 3.2.1 Implementation details

All experiments were conducted under a unified training and evaluation protocol to ensure fair comparison and reproducibility. The experimental environment was based on Ubuntu 22.04, PyTorch 2.1.0, and an NVIDIA Tesla T4 GPU. YOLOv11n initialized with official pretrained weights was adopted as the baseline detector (Jocher and Qiu, 2024), and the random seed was fixed to 42 unless otherwise stated.

Although more recent variants such as YOLOv12 (Tian et al., 2025) and YOLOv13 (Lei et al., 2025) had been released by the time of these experiments, YOLOv11n was adopted as the baseline for three reasons. First, YOLOv11n has reached production-level maturity, with stable open-source releases, well-supported edge-deployment toolchains (TensorRT, ONNX, CoreML), and extensive third-party reproductions—an important practical consideration for an agricultural robotics study in which deployment reproducibility is itself a contribution.

Second, the proposed modules target the C3k2 topology and the FPN–PAN neck organization of YOLOv11; porting them to the area-attention backbone of YOLOv12 or the hypergraph-enhanced head of YOLOv13 would require non-trivial architectural adjustments that confound the ablation analysis. Third, **Table 2** already includes direct comparisons with YOLOv12n and YOLOv13n, against which the proposed method retains advantages of 5.4% and 4.7% mAP@50, respectively; the improvement therefore does not hinge on the baseline generation. The inter-generational gap between YOLOv11n and YOLOv12n on this task is itself only 0.8% mAP@50, indicating that attention-centric architectural changes in YOLOv12 provide limited benefit for dense small-fruit detection.

**Table 2:** Comparison with state-of-the-art methods on the Orchard Pear, Minne Apple, and Mango datasets.

Model	Year	Params Type GFlops (M)				mAP @50	mAP @50:95	APs	ΔmAP @50	FPS
Orchard Pear										
RT-DETR (Zhao et al., 2024)	2023	End-to-End		110.00	32.00	87.60	45.30	24.10	-1.40	86
YOLOv11n (Jocher and Qiu, 2024)	2024	One-Stage		6.30	2.60	89.00	47.50	28.30	—	218
YOLOv12n (Tian et al., 2025)	2025	One-Stage		6.50	2.60	89.80	48.30	29.40	+0.80	195
YOLOv13n (Lei et al., 2025)	2025	One-Stage		6.40	**2.50**	90.50	49.10	30.20	+1.50	188
Rose-Mamba-										
YOLO (You et al., 2025)	2025	Improved		8.20	3.10	91.30	49.80	31.50	+2.30	156
VM-YOLO (Wang et al., 2025a)	2025	Improved		7.80	2.90	90.80	49.40	30.80	+1.80	168
YOLO-CSB (Pan et al., 2026)	2026	Improved		7.10	2.80	91.60	50.20	32.10	+2.60	172
**Ours**	**——**	**Improved**		**5.60**	2.56	**95.20**	**54.60**	**38.20**	**+6.20**	162
Minne Apple										
RT-DETR (Zhao et al., 2024)	2023	End-to-End	110.00		32.00	56.80	30.50	12.80	-3.40	82
YOLOv11n (Jocher and Qiu, 2024)	2024	One-Stage	6.30		2.60	60.20	33.50	15.60	—	212
YOLOv12n (Tian et al., 2025)	2025	One-Stage	6.50		2.60	61.40	34.10	16.30	+1.20	190
YOLOv13n (Lei et al., 2025)	2025	One-Stage	6.40		2.50	62.80	35.00	17.10	+2.60	183
Rose-Mamba-										
YOLO (You et al., 2025)	2025	Improved	8.20		3.10	64.50	36.20	18.40	+4.30	150
VM-YOLO (Wang et al., 2025a)	2025	Improved	7.80		2.90	63.70	35.60	17.80	+3.50	162
YOLO-CSB (Pan et al., 2026)	2026	Improved	7.10		2.80	65.10	36.80	19.20	+4.90	166
**Ours**	**——**	**Improved**	**5.60**		2.56	**77.90**	**45.90**	**28.50**	**+17.70**	156
Mango										
RT-DETR (Zhao et al., 2024)	2023	End-to-End	110.00		32.00	89.50	55.80	34.60	-3.00	89
YOLOv11n (Jocher and Qiu, 2024)	2024	One-Stage	6.30		2.60	92.50	59.10	38.50	—	221
YOLOv12n (Tian et al., 2025)	2025	One-Stage	6.50		2.60	93.20	60.30	39.40	+0.70	198
YOLOv13n (Lei et al., 2025)	2025	One-Stage	6.40		2.50	93.80	61.20	40.10	+1.30	191
Rose-Mamba-										
YOLO (You et al., 2025)	2025	Improved	8.20		3.10	94.50	62.40	41.60	+2.00	159
VM-YOLO (Wang et al., 2025a)	2025	Improved	7.80		2.90	94.10	61.80	40.90	+1.60	171
YOLO-CSB (Pan et al., 2026)	2026	Improved	7.10		2.80	94.80	63.00	42.30	+2.30	175
**Ours**	**——**	**Improved**	**5.60**		2.56	**98.20**	**68.60**	**48.70**	**+5.70**	165

Bold values indicate the best result per column within each dataset block. For Params (M) and GFLOPs, smaller is better; for mAP@50, mAP@50:95, APs, FPS and ΔmAP@50, larger is better. Rows marked “Ours” correspond to the proposed method.

Each model was trained for 120 epochs, with Mosaic augmentation disabled during the last 10 epochs to improve the consistency between training and inference distributions. The input resolution was fixed at 640*imes*640, and the batch size was set to 16. Optimization was performed using SGD. For the proposed modules, the main hyperparameter settings were as follows: the SGEAE module employed 4 heterogeneous experts with Top-K=2 sparse activation; the state dimension of CSP-SGLFE was set to 64; the cyclic shift step of CSDU was set to 1; the parallel kernel sizes in MSCRB were {1, 3, 5}; and the auxiliary scaling range of SOA-IoU was set to 0.5–1.0.

For efficiency evaluation, the number of parameters and GFLOPs were computed using the thop library. Inference speed in terms of FPS was measured on the NVIDIA Tesla T4 GPU with batch size=1. To ensure fairness, all comparison methods reported in this paper were re-trained and re-evaluated using their official open-source implementations under the same experimental environment, including identical hardware conditions, dataset splits, training epochs, and augmentation strategies.

#### 3.2.2 Evaluation metrics

We adopted the standard COCO evaluation protocol for object detection. The primary metrics include Precision (P), which measures the proportion of true positives among predicted positives, and Recall (R), which measures the proportion of ground-truth objects that are successfully detected. Detection accuracy was mainly evaluated by mAP@50, which denotes the mean average precision at an IoU threshold of 0.50, and mAP@50:95, which averages AP over IoU thresholds from 0.50 to 0.95 with a step size of 0.05. Compared with mAP@50, mAP@50:95 imposes a stricter requirement on localization quality and therefore better reflects bounding-box regression accuracy. For the ablation study of SOAIoU, we further report the standard COCO scale-specific metrics, including AP_S_ for small objects with area *<* 32^2^ pixels, AP_M_ for medium objects with area between 32^2^ and 96^2^ pixels, and AP_L_ for large objects with area *>* 96^2^ pixels. In addition, to better reflect orchard-specific detection difficulty, we report two scenario-oriented indicators: *AP*_Dense_ for densely distributed targets and *AP*_Occluded_ for occluded targets. Model efficiency was evaluated using Params (M), GFLOPs, and FPS, thereby jointly assessing detection accuracy, computational complexity, and deployment efficiency. In addition, for the scenario-wise analysis in Section 3.3, we report the false-detection rate (*FDR* = *FP/*(*FP* +*TP*)) and the missed-detection rate (*MDR* = *FN/*(*FN* + *TP*)) under the IoU threshold of 0.5, which directly reflect the severity of spurious detections and omissions in orchard scenes.

### 3.3 Qualitative and quantitative evaluation under complex Orchard scenarios

**Figure 8:**
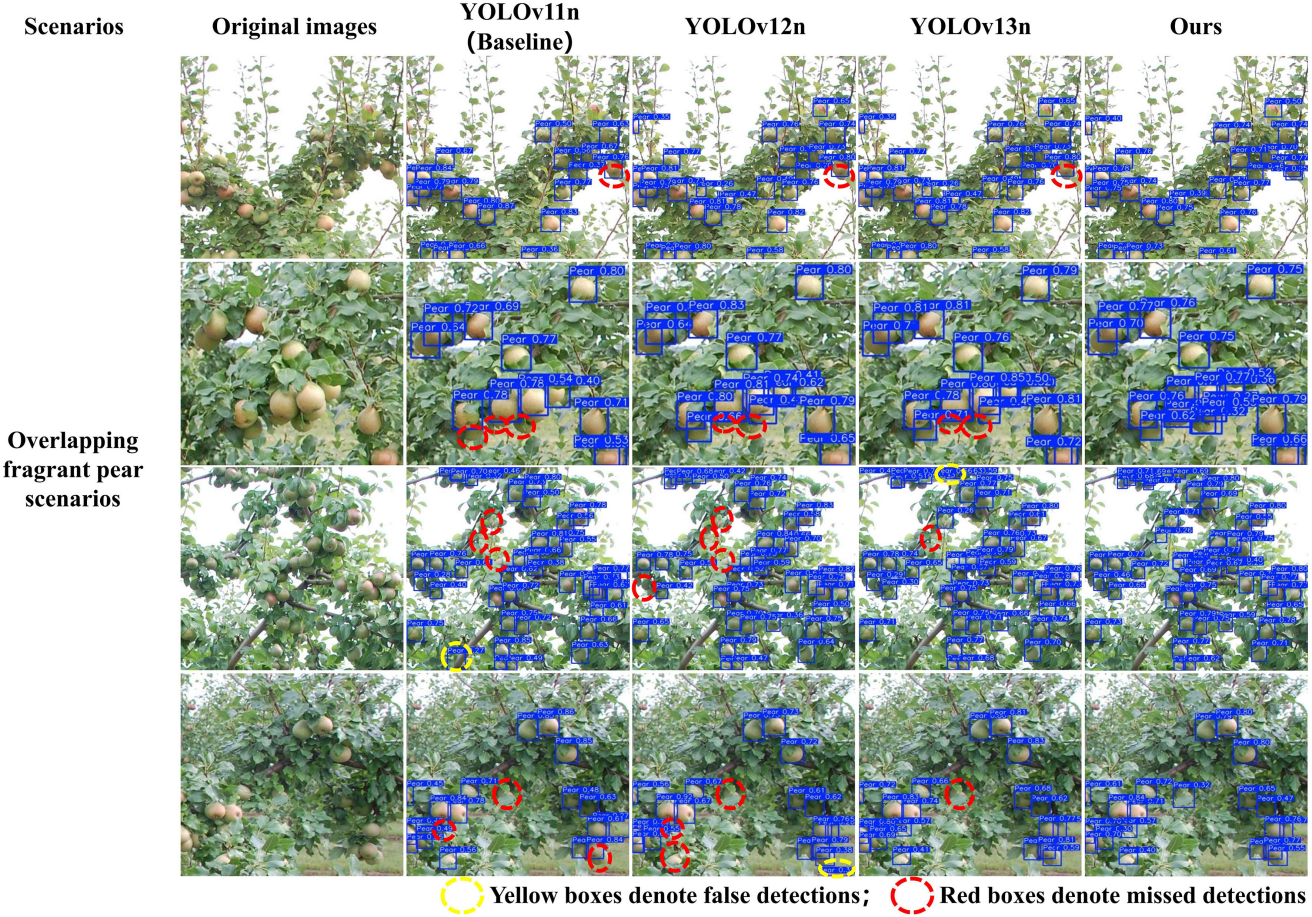
Qualitative results under dense fruit overlap on the Orchard Pear dataset.

**Figure 9:**
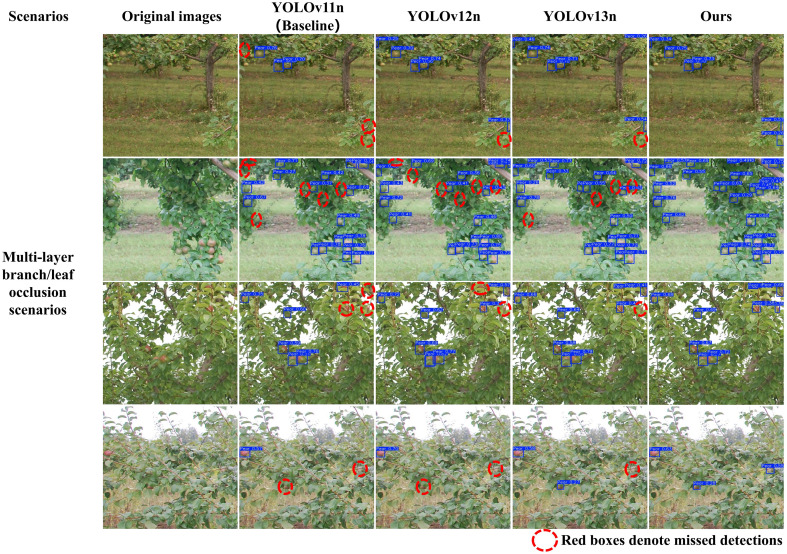
Qualitative results under branch-leaf occlusion on the Orchard Pear dataset.

**Figure 10:**
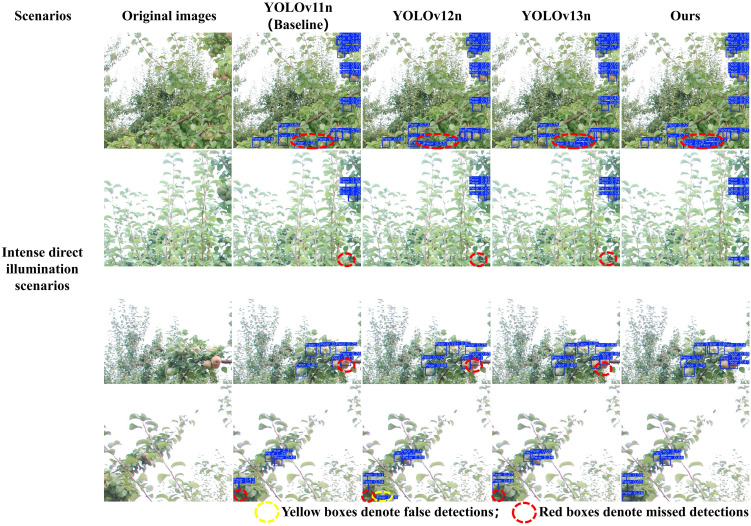
Qualitative results under challenging illumination on the Orchard Pear dataset.

To assess detection performance under realistic field conditions, this section combines quantitative scenario-wise metrics with qualitative visualizations. The Orchard Pear test set is partitioned into three scenario-specific subsets: dense small-scale targets, multilayer branch–leaf occlusion, and intense direct illumination with backlighting. For each subset, Precision, Recall, mAP@50, mAP@50:95, false-detection rate (FDR), and missed-detection rate (MDR) are reported alongside representative detection results. The proposed method is compared with the YOLOv11n baseline and two recent variants, YOLOv12n and YOLOv13n. Quantitative results are summarized in **Table 3**, and qualitative comparisons are shown in **Figures 8**-**10**. Relative to YOLOv13n, mAP@50 increases by 8.60, 6.90, and 6.40 points on the three subsets, and mAP@50:95 increases by 8.90, 7.40, and 6.50 points, respectively. The false-detection rate is more than halved on all three subsets, while the missed-detection rate is reduced by roughly 47–50%.

**Table 3:** Quantitative comparison under three challenging orchard scenarios on the Orchard Pear dataset.

Scenario	Method	Precision	Recall	mAP @50	mAP @50:95	False detection rate	Missed detection rate
Dense Small-scale	YOLOv11n YOLOv12n YOLOv13n	68.50 69.40 70.10	86.30 87.00 87.60	74.20 75.10 75.90	28.30 28.80 29.30	13.80 12.90 12.20	13.50 12.80 12.20
	Ours	76.80	93.20	84.50	38.20	5.60	6.50
Branch-Leaf Occlusion	YOLOv11n YOLOv12n YOLOv13n	73.40 74.10 74.70	91.80 92.30 92.70	82.30 83.00 83.60	39.80 40.50 41.10	10.20 9.40 8.80	9.60 9.00 8.50
	Ours	80.20	96.50	90.50	48.50	4.20	4.50
Intense Illumination	YOLOv11n YOLOv12n YOLOv13n	74.20 75.00 75.60	92.70 93.20 93.60	83.80 84.60 85.30	41.20 41.90 42.60	9.30 8.50 7.90	7.80 7.10 6.60
	Ours	80.80	97.10	91.70	49.10	3.60	3.30

FDR denotes the false-detection rate and MDR denotes the missed-detection rate. Bold values indicate the best result in each column within each scenario block.

#### 3.3.1 Detection under dense small-scale target scenarios

Dense small-scale targets are a major source of detection errors in orchard scenes. Small pears located in the distant canopy provide limited spatial resolution and weak texture cues, which leads to frequent missed detections. Representative samples containing densely distributed small targets are shown in **Figure 8**.

The baseline YOLOv11n omits a considerable number of small fruits, particularly those in the rear canopy or near the image boundary. YOLOv12n and YOLOv13n recover more moderately sized small targets, but systematic omissions remain for the smallest instances. The proposed method identifies a larger proportion of small fruits in the same regions. This outcome is consistent with CSDU and MSCRB, which preserve fine-grained spatial details, and with SOA-IoU, which stabilizes regression for small targets. On this subset, mAP@50 reaches 84.50% and mAP@50:95 reaches 38.20%, with the false-detection rate reduced from 12.20% to 5.60%.

#### 3.3.2 Detection under multi-layer branch–leaf occlusion scenarios

Multi-layer branch–leaf occlusion reduces the visible area of the target and weakens local appearance cues, which increases the difficulty of reliable detection. Representative samples with heavy occlusion are shown in **Figure 9**.

When only a small portion of a fruit is visible, the baseline YOLOv11n produces a considerable number of missed detections, especially for targets simultaneously occluded by multiple branches or leaves. YOLOv12n and YOLOv13n improve recall on moderately occluded targets, but omissions persist in narrow and cluttered regions. The proposed method identifies heavily occluded fruits more consistently. The hierarchical state-space modeling in CSP-SGLFE provides long-range contextual priors, while SGEAE activates different transformation paths according to the occlusion pattern. On this subset, mAP@50 reaches 90.50% and mAP@50:95 reaches 48.50%, with the false-detection rate reduced from 8.80% to 4.20%.

#### 3.3.3 Detection under intense direct illumination and backlighting scenarios

Intense direct illumination and backlighting introduce local overexposure on fruit surfaces and reduce contrast in shadowed regions, which distorts the color and texture cues used by RGB-based detectors. Representative samples under these conditions are shown in **Figure 10**.

The baseline YOLOv11n produces false detections in overexposed regions and missed detections in strongly backlit regions. YOLOv12n and YOLOv13n show improved tolerance to illumination variation, but still fail on targets in low-contrast areas. The proposed method maintains more stable detection across both overexposed and backlit regions. The gain is consistent with the role of CSP-SGLFE, which reduces the dependency on local appearance through global contextual priors, and with SOA-IoU, which stabilizes regression for partially visible targets. On this subset, mAP@50 reaches 91.70% and mAP@50:95 reaches 49.10%, with the false-detection rate reduced from 7.90% to 3.60%.

### 3.4 Ablation studies

#### 3.4.1 Overall ablation study

To characterize both the effectiveness of the proposed framework and the interplay among its four modules, we report the ablation results on the Orchard Pear dataset from three complementary perspectives: individual contribution, progressive integration, and inter-module interdependence. This structure makes visible not only what each module contributes individually, but also how the modules interact when composed. The results are presented in **Table 4**.

**Table 4:** Ablation study on the Orchard Pear dataset, organized into four complementary views to highlight individual contribution, progressive integration, and inter-module interdependence.

#	YOLOv11n	CSP-SGLFE	SGEAE	CSDU	SOA-IoU	P	R	mAP@50	mAP@50:95	APs	Param (M)	FPS
Baseline												
1	✓	×	×	×	×	75.90	97.00	89.00	47.50	28.30	2.60	218
Individual contribution (each module added alone)												
2	✓	✓	×	×	×	75.80	98.00	92.90	51.50	32.50	2.50	184
3	✓	×	✓	×	×	74.00	98.00	92.50	52.10	33.80	1.80	182
4	✓	×	×	✓	×	76.30	97.00	91.90	51.40	35.90	2.70	201
5	✓	×	×	×	✓	76.12	98.00	91.20	49.15	31.80	2.60	211
*Progressive integration (pipeline order: representation* → transformation → fusion → *optimization)*												
6	✓	✓	✓	×	×	76.20	98.00	93.70	53.10	35.40	2.42	172
7	✓	✓	✓	✓	×	77.15	97.85	94.60	53.15	36.50	2.56	163
8	✓	✓	✓	✓	✓	79.21	98.00	95.20	54.60	38.20	2.56	162
Module removal from the full model (interdependence check)												
9	✓	×	✓	✓	✓	78.20	98.00	94.00	53.00	37.10	2.66	168
10	✓	✓	×	✓	✓	76.10	98.00	93.50	52.20	34.80	2.64	176

The CSDU column denotes the joint CSDU+MSCRB neck-fusion module, which is enabled or disabled as a single unit. SOA-IoU is a regression loss and therefore changes neither the parameter count nor FPS, so rows 7 and 8 share the same Param and essentially the same FPS. Because the modules reshape intermediate channel dimensions, parameter counts are *not* additive across the standalone (rows 2–5), progressive (rows 6–8), and removal (rows 9–10) views; the marginal parameter effect of a module therefore differs between its standalone measurement and the progressive or removal configurations.

Individual contribution. When each module is added in isolation to the YOLOv11n baseline (rows 2–5), all four produce positive and non-trivial gains, confirming that none depends on the presence of another for its primary function. CSP-SGLFE yields the largest single-module gain, improving mAP@50 by 3.9%. This is consistent with its role of providing selective global–local context in the backbone: feature representation is the earliest bottleneck in a lightweight detector, so strengthening it benefits all subsequent stages. SGEAE contributes a comparable gain of 3.5%, with its largest improvement concentrated on mAP@50:95 (+4.6%), indicating that input-adaptive transformation primarily sharpens localization rather than gross detection. CSDU+MSCRB contributes 2.9% on mAP@50 but produces the strongest gain on APs (+7.6%), reflecting its role in preserving fine details during multi-scale fusion—the stage at which small-object information is most easily eroded. SOA-IoU contributes 2.2%, the smallest single-module gain, which is expected because regression-level optimization operates on already-extracted features and its effect is upper-bounded by the representation quality upstream.

Progressive integration. To examine whether each module remains effective once the preceding ones are in place, we follow the pipeline order representation → transformation → fusion → optimization (rows 1→2→6→7→8). The cumulative trajectory of mAP@50 is 89.0% → 92.9% → 93.7% → 94.6% → 95.2%, with marginal contributions of +3.9%, +0.8%, +0.9%, and +0.6%, respectively. Two patterns are worth noting. First, every marginal contribution remains strictly positive, indicating that no later-stage module becomes redundant once earlier modules are active. Second, the magnitudes decrease monotonically. This pattern is expected: sequentially added modules address partially overlapping failure cases, so CSP-SGLFE resolves the largest share of recognition errors first and leaves successively smaller but still distinct failure modes for later modules. The fact that SOA-IoU still contributes +0.6% at the final stage, despite acting only on the regression objective, confirms that localization-level optimization retains measurable value even when the feature pipeline is already strong.

Inter-module interdependence. Two further observations characterize how the modules interact. First, the sum of the four individual gains is 12.5% (3.9 + 3.5 + 2.9 + 2.2), whereas the full-model gain over the baseline is 6.2%. Defining an overlap coefficient as $1-{\text{Δ}}_{\text{full}}/\sum_{i}{\text{Δ}}_{i}$, we obtain approximately 50% overlap, which quantifies the degree to which the four modules address shared failure modes. This level of overlap is anticipated under the selective information propagation formulation, because all four modules share a common principle of suppressing non-informative signals, even though they act at different stages of the pipeline. The remaining half of the individual gains, captured only when the modules are composed, reflects the genuinely complementary contributions that drive the final accuracy. Second, the two module-removal configurations (rows 9–10) show that removing CSP-SGLFE or SGEAE from the full model reduces mAP@50 to 94.0% and 93.5%, respectively, both of which still exceed any single-module configuration. This indicates that the framework does not rely on a single load-bearing module, and that each removal leaves a degraded but still functional version of the system. These observations indicate that the four modules are neither interchangeable (in which case the overlap coefficient would approach 100%) nor independent (in which case it would approach 0%); they occupy partially overlapping roles at the four pipeline stages formalized in Section 2.1. Overall, the three perspectives jointly establish that CSP-SGLFE, SGEAE, CSDU+MSCRB, and SOA-IoU act on distinct stages of the detection pipeline—contextual representation, adaptive transformation, multi-scale fusion, and regression optimization—with sufficient complementarity to drive the +6.2% mAP@50 improvement over the baseline, and sufficient redundancy that the framework degrades gracefully rather than catastrophically under partial ablation.

#### 3.4.2 Scenario-specific performance analysis

To quantitatively assess how each component addresses the three major challenges introduced in Section 1, we further partitioned the Orchard Pear test set into scenario-specific subsets and reported mAP@50:95 on occluded, small-scale, and illumination-degraded scenes. The results are summarized in **Table 1**.

The decomposition clearly reflects the functional role of each module. CSP-SGLFE achieves the largest gain on the illumination-degraded subset, improving mAP@50:95 by 5.6 percentage points over the baseline (41.20% → 46.80%), which confirms that selective global–local context modeling is particularly effective when local appearance cues are corrupted by strong lighting variation. SGEAE yields the largest gain on the branch-leaf occlusion subset, with an improvement of 6.4 percentage points (39.80% → 46.20%), indicating that input-adaptive sparse routing dynamically allocates specialized transformation pathways to handle the heterogeneous visibility patterns introduced by multilayer occlusion. CSDU+MSCRB achieves its largest gain on the dense small-scale subset, improving mAP@50:95 by 7.6 percentage points (28.30% → 35.90%), which is consistent with its design goal of preserving fine structural details through parameter-free directional channel mixing and multi-branch depthwise receptive fields—the features most vulnerable to loss in conventional top-down interpolation. SOA-IoU provides stable improvements across both the branch-leaf occlusion (+4.0) and dense small-scale (+3.5) subsets, verifying the effectiveness of combining shape-prior constraints with scale-aware gradient allocation for hard regression samples.

The full model obtains the best result across all subsets, reaching 38.2% on the dense small-scale, 48.5% on the branch-leaf occlusion, and 49.1% on the illumination subset. These results confirm that the four modules address distinct degradation factors— illumination robustness via CSP-SGLFE, occlusion adaptability via SGEAE, small-object detail preservation via CSDU+MSCRB, and hard-sample regression via SOA-IoU—and together cover the principal challenges of unstructured orchard scenes.

#### 3.4.3 Cross-dataset generalization ablation study

To investigate whether the benefits of the proposed framework generalize beyond the self-constructed Orchard Pear dataset, we further conducted cross-dataset ablation experiments on Minne Apple and Mango. The results are reported in **Supplementary Appendix Table 13**.

On Minne Apple, the complete model improves mAP@50 and mAP@50:95 by 17.7% and 12.4%, respectively, over the baseline; on Mango, the corresponding gains are 5.7% and 9.5%. On both datasets every module contributes a positive gain when added in isolation, and the modules combine into a substantial cumulative improvement, which suggests that their usefulness is not confined to the Orchard Pear distribution.

The larger margin on Minne Apple reflects the starting point rather than a change in which modules matter. The per-module ordering is in fact the same as on Orchard Pear: CSP-SGLFE again provides the largest single gain (+3.3 mAP@50), while the fusion and regression modules contribute the least (+1.3 and +1.5), consistent with representation being the earliest bottleneck. What differs is the baseline: as noted in Section 3.6.1, the YOLOv11n baseline begins from an unusually low score on Minne Apple, which leaves more headroom for any well-designed method to recover. The pairwise margins over recent lightweight detectors in **Table 2** are therefore a more conservative estimate of the gain attributable to our modules than the raw improvement over the baseline.

These cross-dataset results indicate that the proposed framework transfers across different fruit categories, density levels, and visual characteristics rather than being tailored to the Orchard Pear dataset alone.

### 3.5 Grad-CAM visualization of cross-dataset generalization

**Figure 11:**
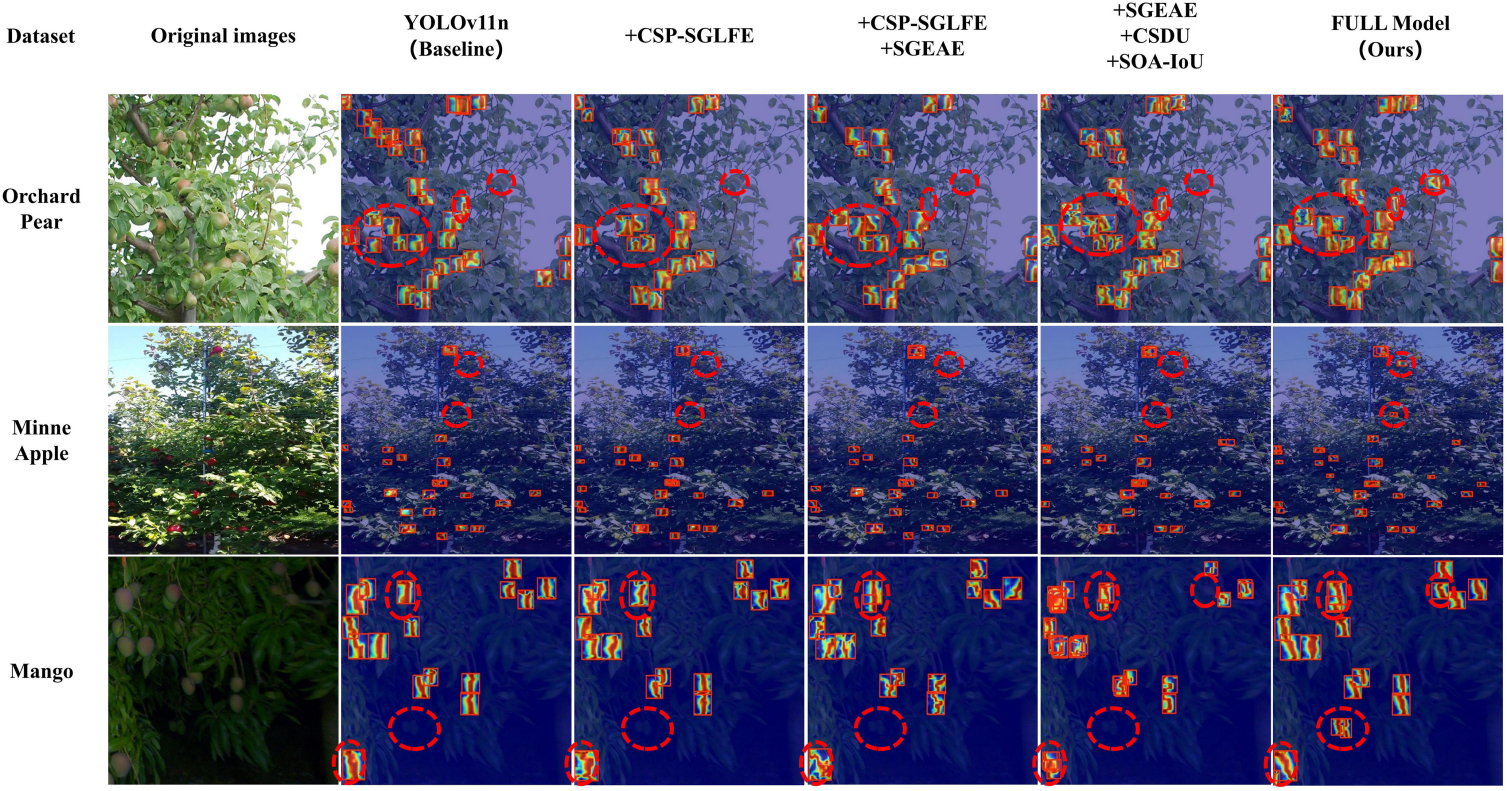
Cross-dataset Grad-CAM visualization for generalization analysis.

To further examine whether the feature enhancement effects of the proposed framework remain consistent across different orchard datasets, we extended the Grad-CAM analysis to Minne Apple and Mango. The visualization results are shown in **Figure 11**.

On Orchard Pear, the baseline model exhibits noticeable background leakage, whereas the proposed full model constrains activation more effectively to fruit regions. On Minne Apple, where fruits are extremely dense and relatively small, the baseline produces diffuse response maps that struggle to separate adjacent instances. As the proposed modules are progressively added, the activation patterns become increasingly concentrated and better separated, and the full model shows clearer instance-level responses for densely distributed apples.

On Mango, where low color contrast between fruits and leaves makes foreground background discrimination more difficult, the proposed framework produces sharper and more localized responses around fruit regions than the baseline. These observations suggest that the proposed modules do not merely adapt to a single data distribution, but instead provide a more transferable feature enhancement mechanism across different fruit morphologies and orchard conditions.

Quantitative evaluation of attention quality. To complement the qualitative GradCAM observations with quantitative measurements, we computed two established metrics that quantify the alignment between model attention and ground-truth object locations. The first is the Pointing Game accuracy (Zhang et al., 2018), defined as the fraction of test images for which the argmax of the Grad-CAM map falls inside any ground-truth bounding box. The second is the Attention-IoU (A-IoU), computed as the IoU between the binarized Grad-CAM mask—obtained by thresholding each map at the top 30% of activation values—and the union of ground-truth bounding boxes. Both metrics are standard in the weakly-supervised localization literature, and higher values indicate that the model’s attention is more tightly aligned with the objects that should be detected. As reported in **Supplementary Appendix Table 16**, the YOLOv11n baseline achieves 71.3% Pointing Game accuracy and 0.342 A-IoU; with the proposed modules progressively integrated, these values rise to 84.7% and 0.518 for the full model, representing relative improvements of +18.8% and +51.5%, respectively. The monotonic increase at each integration stage shows that each of the four modules contributes to more object-grounded attention rather than to spatially diffuse or background-biased activations, confirming the qualitative patterns observed in **Figure 11** with quantitative support.

### 3.6 Comparison with state-of-the-art methods

#### 3.6.1 Comparison with state-of-the-art methods on three benchmark datasets

The quantitative comparison on Orchard Pear, Minne Apple, and Mango is reported in **Table 2**.

On Orchard Pear, the proposed method achieves 95.2% mAP@50 and 54.6% mAP@50:95 with the smallest computational cost (5.60 GFLOPs) among all compared detectors and 2.56 M parameters, second only to YOLOv13n (2.50 M) by 0.06 M. Compared with the recent lightweight detector YOLO-CSB, our method improves mAP@50 and mAP@50:95 by 3.6% and 4.4%, respectively, while using 21% fewer GFLOPs and 9% fewer parameters. Among end-to-end transformer detectors we retain RT-DETR as the representative real-time baseline, since the earlier DETR (Carion et al., 2020) and Deformable-DETR (Zhu et al., 2021), with parameter counts above 40 M, lie outside the lightweight regime considered here. Relative to RT-DETR, the proposed method improves mAP@50 by 7.6% while requiring only 8.0% of its parameters and 5.1% of its GFLOPs. These results indicate that the proposed selective information propagation framework offers a clear advantage for lightweight embedded deployment in orchard environments.

On the Minne Apple dataset (**Table 2**), the proposed method reaches 77.9% mAP@50, corresponding to a 17.7% absolute gain over the YOLOv11n baseline. This gap should be decomposed into two distinct contributions before it is interpreted as generalization. First, the YOLOv11n baseline itself starts from an unusually low 60.2% mAP@50 on Minne Apple—substantially lower than its performance on our Orchard Pear dataset (89.0%)— which leaves considerable headroom for any reasonably designed method to improve upon. A stronger baseline choice would therefore be expected to close part of the reported 17.7% gap. Second, after controlling for baseline headroom by comparing directly with the strongest lightweight detectors on the same Minne Apple evaluation, our method still exceeds YOLO-CSB (Pan et al., 2026) and Rose-Mamba-YOLO (You et al., 2025)—the two best-performing baselines in **Table 2**—by 12.8 and 13.4 percentage points of mAP@50, respectively. These pairwise margins against recent state-of-the-art baselines, rather than the 17.7% relative to the weakest baseline, should be taken as the more reliable estimate of cross-scene generalization attributable to the proposed modules. A complementary feature-level analysis of the distribution shift between the source and target datasets is reported in Section 3.6.2. Based on this decomposition, the proposed method therefore offers a consistent but moderate cross-scene advantage on the order of approximately 3–13% mAP@50 over the strongest lightweight baselines across the datasets tested, with the precise magnitude depending on how much headroom the reference baseline retains. The Mango dataset result (5.7% gain over the YOLOv11n baseline, and 3.4% over the strongest baseline YOLO-CSB) reflects a scenario closer to the saturated end of this range.

On Mango, where the baseline performance is already relatively high, the proposed method still achieves 98.2% mAP@50 and 68.6% mAP@50:95, corresponding to gains of 5.7% and 9.5% over YOLOv11n, respectively. This indicates that the proposed framework remains effective even when the target scale is generally larger and the main challenge shifts toward low foreground–background contrast rather than extreme density. The consistent gains across all three datasets confirm that the proposed method is not limited to a specific fruit category or scene distribution, but exhibits strong cross-scene generalization.

Beyond the end-to-end detectors compared in **Table 2**, a complementary question is whether the gains obtained over YOLOv11n can be attributed to the proposed selective-propagation modules themselves, or alternatively to a particularly fortunate choice of lightweight building blocks. To disentangle these two factors, we conducted a component level comparison in which either the backbone or a specific module of YOLOv11n is replaced with a lightweight counterpart drawn from the mobile-CNN, mobile-Transformer, and agriculture-specific literature, while keeping the rest of the pipeline and training protocol identical. This controlled replacement design isolates the marginal effect of each lightweight component under a fair training regime. The full results of the six variants, together with a re-implemented FruitDet (Kateb et al., 2021) as a representative agriculture-specific detector, are reported in **Supplementary Appendix Table 15**. Across all tested variants, our method retains a consistent accuracy advantage on the Orchard Pear dataset. We note that AgriDet (Pal et al., 2023), sometimes listed alongside FruitDet in agricultural-detection surveys, is a plant-leaf-disease severity classification framework based on INC-VGGN rather than an object detector, and is therefore not applicable as a direct baseline in this setting.

From the perspective of the accuracy–efficiency trade-off, the proposed detector compares particularly favorably with recent lightweight one-stage and improved models. While several competing methods achieve moderate accuracy gains by increasing model complexity, the proposed method simultaneously reduces computational burden and improves detection robustness. This advantage stems from the coordinated selective design across representation, transformation, fusion, and optimization, rather than from simply enlarging network capacity. Therefore, the proposed framework provides a more effective and deployment-friendly solution for fruit detection in unstructured orchards.

#### 3.6.2 Feature distribution shift analysis

The sizable performance gap between the Pear→Minne Apple and Pear→Mango transfer settings raises a natural question: is the proposed method genuinely capturing transferable fruit-detection cues, or does the observed gap reflect only differences in baseline headroom? To probe this question at the feature level, we quantified the distribution shift between the training source (Orchard Pear) and the two evaluation targets using the Maximum Mean Discrepancy (MMD) with a Gaussian kernel (Gretton et al., 2012). Specifically, we extracted P4-level backbone features {*f_i_*} from 500 randomly sampled images per dataset and computed the squared MMD as defined in Equation 30:

(30) $${\text{MMD}}^{2}(D_{s},D_{t})=\frac{1}{n_{s}^{2}}\underset{i,j}{\sum }k(f_{i}^{s},f_{j}^{s})+\frac{1}{n_{t}^{2}}\underset{i,j}{\sum }k(f_{i}^{t},f_{j}^{t})-\frac{2}{n_{s}n_{t}}\underset{i,j}{\sum }k(f_{i}^{s},f_{j}^{t}),$$

where *k*(·, ·) is the Gaussian RBF kernel with bandwidth set to the median pairwise distance of the source set, and *n_s_*= *n_t_*= 500. The resulting MMD^2^ values are 0.12 for Pear→Mango and 0.30 for Pear→Minne Apple, with the Pear→Minne Apple shift substantially larger. This ordering is consistent with qualitative observation—Mango shares near-rounded fruit geometry and tropical canopy structure that partially overlap with pear orchards, while Minne Apple contains densely clustered, small-scale apple instances that differ markedly from the Korla pear acquisition conditions. We next examine how the measured distribution shift relates to the cross-dataset accuracy gap discussed in Section 3.6.1. Two caveats apply before interpretation. First, as acknowledged in Section 3.6.1, the YOLOv11n baseline itself leaves more headroom on Minne Apple (60.2% mAP@50) than on Mango (92.5%), so a portion of the 17.7% raw gain on Minne Apple reflects this headroom rather than generalization per se. Second, baseline headroom and feature-level distribution shift are not independent: when a target domain is farther from the source, all detectors trained on the source are expected to degrade more, so large MMD^2^ and large headroom can co-occur. The MMD analysis therefore cannot by itself disentangle these two factors.

What the MMD analysis can establish is that the backbone features extracted by our method do not collapse under the larger Pear→Minne Apple shift in a way that would eliminate its relative advantage over recent lightweight baselines. Specifically, on the dataset with MMD^2^ = 0.30, our method retains a 12.8–13.4 percentage-point lead over YOLO-CSB and Rose-Mamba-YOLO, whose own architectures are trained under the same Orchard Pear source distribution; since the headroom argument applies equally to these competitors, the relative margin is a quantity in which headroom has already been approximately controlled for. We do not claim that this margin is solely attributable to the selective-propagation design—no experiment short of full cross-domain training could establish that—but the feature-level evidence (MMD^2^) combined with the pairwise margin (12.8–13.4%) is jointly more informative than either quantity alone.

We emphasize that our framework contains no explicit domain-adaptation components; the observed behavior reflects the in-distribution representational properties of the selective propagation principle.

### 3.7 Deployment feasibility analysis on edge platforms

In addition to detection accuracy, practical orchard deployment requires low latency, high throughput, and energy-efficient inference on embedded hardware. To evaluate these aspects, we deployed the proposed model on three representative NVIDIA Jetson platforms: Jetson AGX Orin, Jetson Orin NX, and Jetson Orin Nano. The deployment results under TensorRT 8.6 optimization in MAX power mode are presented in **Table 5**.

**Table 5:** Deployment performance and quantization accuracy on NVIDIA Jetson embedded platforms under TensorRT 8.6 optimization in MAX power mode.

Platform	Precision	Latency (ms)	FPS	Power (W)	Energy/Inf (mJ)	mAP @50	mAP @50:95	ΔmAP @50:95	Peak memory usage
Tesla T4 (Server)	FP32	—	—	—	—	95.20	54.60	—	876 MB
Jetson AGX Orin	FP32	8.6	116.3	22.5	193.5	—	—	—	842 MB
Jetson AGX Orin	FP16	5.2	192.3	23.8	123.8	95.10	54.45	−0.15	486 MB
Jetson AGX Orin	INT8	3.8	263.2	24.1	91.6	94.80	54.05	−0.55	334 MB
Jetson Orin NX	FP32	14.2	70.4	16.8	238.6	—	—	—	856 MB
Jetson Orin NX	FP16	8.7	114.9	17.5	152.3	95.05	54.40	−0.20	498 MB
Jetson Orin NX	INT8	6.1	163.9	17.9	109.2	94.65	53.85	−0.75	346 MB
Jetson Orin Nano	FP32	22.8	43.9	11.2	255.4	—	—	—	874 MB
Jetson Orin Nano	FP16	13.5	74.1	12.0	162.0	94.95	54.30	−0.30	512 MB
Jetson Orin Nano	INT8	9.4	106.4	12.3	115.6	94.40	53.60	−1.00	358 MB

Bold values indicate the best result per column. For Latency, Power and Energy/Inf, smaller is better; for FPS and mAP, larger is better.

On Jetson AGX Orin, the proposed model achieves an inference latency of 5.2 ms in FP16 mode, corresponding to 192.3 FPS. In INT8 mode, the speed further increases to 263.2 FPS, while the energy consumption per inference decreases to 91.6 mJ. These results indicate that the model can provide highly responsive perception on high-performance edge robotic platforms.

On Jetson Orin NX, which represents a more balanced embedded computing platform, the proposed model reaches 114.9 FPS in FP16 mode and 163.9 FPS in INT8 mode, with only minor accuracy degradation. This suggests that the model remains well-suited to real-world deployment even under more constrained computational resources.

On Jetson Orin Nano, the most resource-limited platform among the three, the detector still achieves 74.1 FPS in FP16 mode and 106.4 FPS in INT8 mode. The corresponding energy consumption per inference is only 162.0 mJ and 115.6 mJ, respectively. Even in this highly constrained setting, the inference speed remains substantially above the typical real-time threshold of 30 FPS, indicating strong potential for battery-powered mobile harvesting systems.

Quantization robustness and interpretation. FP16 deployment introduces only minor accuracy degradation across all platforms, with mAP@50:95 decreasing by 0.15%–0.30%. INT8 quantization incurs a higher—but still bounded—cost, with a maximum mAP@50:95 drop of 1.00% observed on Orin Nano. This drop warrants careful interpretation rather than dismissal at face value: for a detector already operating at 95.20% mAP@50 and 54.60% mAP@50:95, the same absolute change represents a proportionally larger residual-error increase, and its practical impact depends on which IoU thresholds the downstream task actually cares about.

The mAP@50:95 metric averages accuracy over IoU thresholds from 0.50 to 0.95, so a 1.00% drop at this aggregate level is not distributed uniformly across thresholds. Low precision arithmetic primarily perturbs the fine-grained regression of box boundaries rather than the presence-or-absence decision of a detection. Quantization-induced error therefore concentrates at stricter IoU thresholds, where correct detections with slightly misaligned edges are counted as misses. At the loose end of the spectrum (mAP@50), the accuracy gap between the FP32 reference and the Orin Nano INT8 deployment is small, because most detections still overlap the ground truth by more than 0.5. At stricter thresholds—where a few pixels of boundary drift are sufficient to flip a true positive to a false positive—the gap grows visibly, and this is the regime in which the aggregate 1.00% mAP@50:95 drop is mostly concentrated.

This decomposition translates into a concrete deployment guideline. For applications in which coarse localization is sufficient—yield estimation, fruit counting, or high-level scene understanding—INT8 on Orin Nano offers the best accuracy–energy trade-off, since the cost at the loose IoU regime that dominates such tasks is minor. For applications in which precise boundary alignment matters—most notably closed-loop robotic grasping, where a few pixels of boundary drift at the image level can translate into grasp misalignment at the arm level—we recommend FP16 on Orin Nano, which preserves mAP@50:95 within 0.30% of the FP32 reference while still sustaining real-time inference (74 FPS at 162 mJ per inference). Jetson AGX Orin provides enough headroom to meet the stricter accuracy requirements even at INT8 precision (263 FPS at 91.6 mJ per inference, with mAP@50:95 dropping only 0.55%), and is therefore the preferred choice for deployment scenarios that cannot tolerate any noticeable boundary degradation.

Beyond accuracy, INT8 quantization reduces peak memory usage by approximately 60%, further confirming suitability for memory-constrained edge deployment without out-of-memory bottlenecks. Taken together, the accuracy–energy–memory trade-off across the three Jetson platforms shows that the proposed model supports a usable operating point at every precision and hardware tier considered, provided that the precision is chosen in line with the application’s IoU-sensitivity profile rather than defaulting to the smallest numeric format available.

## 4 Discussion and conclusion

This study presents a selective information propagation framework to address the bottlenecks of lightweight fruit detection in unstructured orchards. By integrating CSP-SGLFE, SGEAE, CSDU+MSCRB, and SOA-IoU, the proposed method effectively mitigates localization errors caused by dense overlap, severe occlusion, and dynamic lighting. Evaluations across three distinct datasets show that the method improves detection accuracy over existing lightweight models while meeting the real-time and power constraints of edge-computing platforms.

A key finding of this research is that the performance bottleneck in lightweight orchard perception stems less from insufficient network capacity and more from the inefficient allocation of feature representation. In complex environments, simply scaling up model size or depth incurs high computational costs without successfully resolving localized degradations, such as partial occlusion. Instead, our results demonstrate that dynamically routing and selectively enhancing features provides a more viable path for resource constrained agricultural robotics. Furthermore, the model’s consistent cross-dataset performance indicates that the framework learns generalized target characteristics rather than overfitting to specific orchard conditions.

While the proposed method is robust across the scenarios tested, several limitations remain. The three evaluation subsets—dense small-scale, branch–leaf occlusion, and intense illumination—approximate rather than directly isolate the pear-specific factors that motivate the design (chromatic camouflage, mutual boundary occlusion within clusters, and weak shape priors from near-rotational symmetry): illumination acts mainly as a general appearance stressor, and the effect of weak shape priors is observed only indirectly through occluded and small-target performance, so subsets defined along each factor would permit a sharper attribution in future work. A second limitation is that the framework relies solely on 2D RGB imagery, which inherently struggles with extreme depth ambiguity and complete self-occlusion. Incorporating multimodal inputs, such as RGB-D or thermal sensing, could provide essential geometric priors to resolve these edge cases. Additionally, although the detector achieves high efficiency in benchmarking, real-world deployment on field robots introduces further physical complexities, including camera motion blur and concurrent computational loads from navigation systems. Future work will therefore focus on system-level co-optimization. The cross-dataset gains reported here are obtained without any explicit domain alignment; coupling the framework with cross-species subdomain adaptation (Yan et al., 2023) is a natural way to push generalization further under larger source–target shift. We also plan to extend the detection outputs from axis-aligned bounding boxes to instance segmentation and 3D spatial localization, which are crucial prerequisites for precision robotic grasping and automated yield mapping.

In summary, this work contributes an efficient and reasonably general perception baseline for lightweight fruit detection in complex orchard environments.

## Data Availability

The raw data supporting the conclusions of this article will be made available by the authors, without undue reservation.
