# Supplementary material for: Lightweight pear detection in unstructured orchards via selective information propagation
Source: Front Plant Sci. 2026 Jul 13;17:1835001. doi: 10.3389/fpls.2026.1835001 (PMC13402388; doi:10.3389/fpls.2026.1835001)
Supplement: Supplementary file 1 [file DataSheet1.pdf]

Supplementary Material

1 ABLATION STUDY

1.1 Ablation Study on the CSP-SGLFE Module

To verify the effectiveness of the proposed CSP-SGLFE module in enhancing contextual perception and feature discrimination in complex orchard environments, we conducted progressive ablation experiments on its main components. The results are summarized in Table **6**.

Table 6. Ablation study of CSP-SGLFE on the Orchard Pear dataset

Model Configuration	HSM-SSD	CGLU	DWConv	Alpha	mAP@50	mAP@50:95	Param(M)	GFLOPs	FPS
YOLOv11n Baseline (C3k2)	×	×	×	×	89.00	47.50	2.60	6.30	218
C3k2 + HSM-SSD	✓	×	×	×	90.2	48.70	2.52	6.48	195
C3k2 + CGLU	×	✓	×	×	89.8	48.30	2.55	6.38	208
C3k2 + HSM-SSD + CGLU	✓	✓	×	×	91.2	49.60	2.48	6.52	189
C3k2 + HSM-SSD + CGLU + DWConv	✓	✓	✓	×	92.0	50.50	2.49	6.62	185
CSP-SGLFE (Full)	✓	✓	✓	✓	92.90	51.50	2.50	6.70	184

When HSM-SSD is introduced alone, the detector achieves a 1.2% improvement in mAP@50 over the baseline, indicating that efficient global dependency modeling contributes positively to feature representation. When only CGLU is added, mAP@50 increases to 89.8%, showing that gated channel modulation helps emphasize discriminative responses while suppressing irrelevant interference. When HSM-SSD and CGLU are jointly incorporated, mAP@50 further rises to 91.2%, suggesting that global contextual information provides a stronger basis for subsequent selective channel enhancement.

After further introducing the 3×3 depthwise convolution to reinforce local spatial inductive bias, the performance reaches 92.0% mAP@50. Finally, the complete CSP-SGLFE module with adaptive residual scaling achieves 92.9% mAP@50 and 51.5% mAP@50:95, corresponding to gains of 3.9% and 4.0% over the baseline, respectively. These results demonstrate that local modeling, global dependency extraction, and gated feed-forward enhancement are complementary within the proposed block.

In addition, the full CSP-SGLFE module slightly reduces the total number of parameters relative to the baseline, reflecting the parameter efficiency of the state-space modeling design. Although the inference speed decreases from 218 FPS to 184 FPS, this reduction remains acceptable for real-time robotic harvesting applications. Overall, CSP-SGLFE provides a favorable trade-off between accuracy improvement and lightweight deployment efficiency.

1.2 Ablation Study on the SGEAE Module

To evaluate the effectiveness of the proposed SGEAE module for adaptive feature transformation under heterogeneous orchard degradations, we performed systematic ablation experiments on its insertion position, routing mechanism, shared baseline branch, sparsity level, and expert composition. The results are reported in Table **7**.

Table 7. Ablation study and sensitivity analysis of SGEAE on the Orchard Pear dataset.

(a) Ablation study of SGEAE

Model Configuration	SAGR	Shared	Sparse	Position	mAP@50	mAP@50:95	Param(M)	GFLOPs	FPS
YOLOv11n (Baseline)	×	×	×	—	89.00	47.50	2.60	6.30	218
+ SGEAE (P4 only)	✓	✓	✓	P4	90.30	49.00	2.18	5.82	198
+ SGEAE (P3+P4)	✓	✓	✓	P3+P4	91.40	50.50	1.96	5.68	189
+ SGEAE (P3+P4+P5)	✓	✓	✓	P3+P4+P5	92.50	52.10	1.80	5.60	182
A3 w/o SAGR (Adaptive Router)	×	✓	✓	P3+P4+P5	91.30	50.80	1.78	5.52	186
A3 w/o Shared Expert	✓	×	✓	P3+P4+P5	90.70	49.90	1.72	5.48	185
A3 w/ Top-1 (K=1)	✓	✓	K=1	P3+P4+P5	91.60	51.20	1.80	5.46	195
A3 w/ Top-3 (K=3)	✓	✓	K=3	P3+P4+P5	91.10	50.40	1.80	5.74	172
A3 w/ Ghost Experts only	✓	✓	Ghost	P3+P4+P5	92.30	51.80	1.80	5.96	165
A3 w/ Inverted Experts only	✓	✓	Inv	P3+P4+P5	91.70	51.00	1.68	5.38	196

Note: In the Sparse column, K=1/K=3 denotes the Top-K sparsity variants, whereas Ghost/Inv. indicates the expert type variants. The default configuration is N=4 and K=2. The expert pool comprises three heterogeneous types: point-wise experts, Ghost experts, and inverted residual experts.

(b) Sensitivity analysis of N and K

N	K	K/N	mAP@50	mAP@50:95	Params (M)	GFLOPs	FPS
2	1	0.50	90.80	49.70	1.72	5.41	197
4	1	0.25	91.60	51.20	1.80	5.46	195
4	2	0.50	92.50	52.10	1.80	5.60	182
4	3	0.75	91.10	50.40	1.80	5.74	172
4	4	1.00	90.70	49.90	1.80	5.88	164
6	2	0.33	92.30	51.80	2.04	5.88	170
6	3	0.50	92.60	52.00	2.04	6.02	162
6	4	0.67	91.90	51.10	2.04	6.16	156
8	2	0.25	92.00	51.50	2.32	6.22	158
8	4	0.50	92.40	51.90	2.32	6.50	148
8	6	0.75	91.50	50.80	2.32	6.78	140

Note: Highlighted row indicates the default configuration (N=4, K=2). Results for N=4 with $K \in \{1, 2, 3\}$ are drawn from the ablation study; the remaining rows are added for the sensitivity analysis.

Expert utilization analysis. To empirically validate that the dual-stream architecture prevents expert collapse, we analyzed the routing behavior of SGEAE throughout training. Figure **12**(a) shows the per-expert activation frequency at the final training epoch for both the full SGEAE and an ablation variant with the shared stream removed. The full dual-stream configuration maintains balanced utilization across all four experts (frequencies $\{0.27, 0.25, 0.26, 0.22\}$), whereas removing the shared stream causes one expert to dominate 61% of the routing decisions, with the least-used expert receiving only 8%. Figure **12**(b) plots the normalized load-balance coefficient $\mathcal{L}_{\text{bal}}$ over training epochs, showing that the full design converges to and maintains $\mathcal{L}_{\text{bal}} \approx 1.14$, while the variant without the shared stream drifts toward $\mathcal{L}_{\text{bal}} \approx 1.89$ by epoch 60 and remains imbalanced thereafter. This confirms that the shared pathway stabilizes early routing decisions and prevents the self-reinforcing loop that drives collapse.

Complete (N, K) sensitivity sweep. To justify the default setting $N = 4, K = 2$, we performed a systematic sweep over $N \in \{2, 4, 6, 8\}$ and $K \in \{1, \ldots, N\}$. The extended results are presented in Table

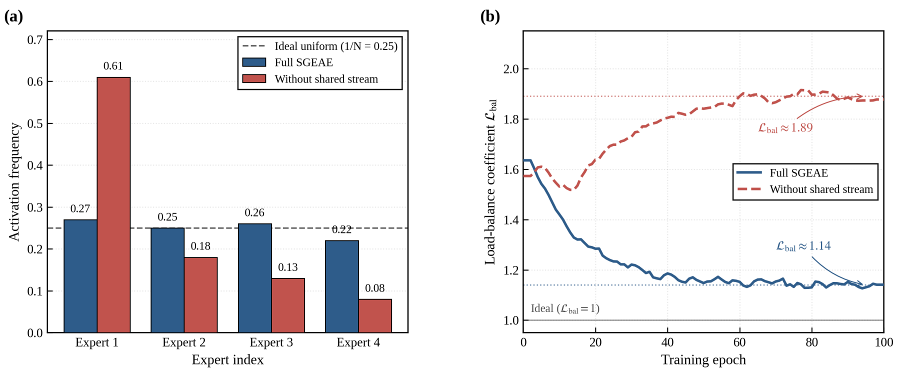

Figure 12. Empirical verification of expert utilization in SGEAE. (a) Per-expert activation frequency histogram at the final training epoch for the full SGEAE (blue bars) and the ablation variant without the shared stream (red bars); the dashed horizontal line indicates the ideal uniform utilization $1/N = 0.25$ for $N = 4$. (b) Normalized load-balance coefficient $\mathcal{L}_{\text{bal}}$ over training epochs; the full SGEAE (blue solid) converges to $\mathcal{L}_{\text{bal}} \approx 1.14$, whereas the variant without the shared stream (red dashed) diverges to $\mathcal{L}_{\text{bal}} \approx 1.89$.

7b. Three observations support the default choice. First, at each value of N, the best performance is obtained near $K/N \approx 0.5$; configurations with $K/N = 0.25$ under-utilize expert diversity, while $K/N \geq 0.75$ introduce redundant computation. Second, increasing N from 4 to 6 yields a marginal gain of $+0.1\%$ mAP@50 while increasing parameters by 13%, and further increases to $N = 8$ actually reduce mAP@50 slightly due to routing dilution across a larger expert pool. Third, the $N = 4$, $K = 2$ configuration achieves the best balance of accuracy, parameter count, and FPS among all tested combinations, confirming it as the Pareto-optimal choice for the deployment targets considered in Section 3.7.

Regarding the insertion position, the performance gain increases progressively as SGEAE is extended from P4 only to P3 + P4 + P5. Specifically, mAP@50 improves from 90.3% to 92.5%, yielding an overall gain of 3.5% over the baseline. This trend indicates that conditional sparse transformation is beneficial across multiple feature levels rather than being limited to a single stage. At the same time, because only a subset of experts is activated under the sparse routing strategy, the actual computational overhead remains controlled.

Component ablation further clarifies the contribution of each design element. Removing the spatially aware gating router leads to a 1.2% drop in mAP@50, suggesting that convolution-based routing preserves structural cues more effectively than fully connected token-level gating. Removing the shared expert branch causes an even larger drop of 1.8%, indicating that the shared-sparse dual-stream design plays an important role in stabilizing transformation behavior and avoiding expert collapse during training.

We also investigated the effect of routing sparsity. The default setting Top-K=2 yields the best overall performance. Using K=1 leads to insufficient utilization of expert diversity and reduces accuracy by 0.9%, whereas K=3 introduces redundant computation and lowers accuracy by 1.4%. In addition, the heterogeneous expert pool consistently outperforms homogeneous expert configurations composed only of Ghost experts or only of inverted residual experts. This result indicates that diverse transformation paths are more suitable for handling the varied degradation patterns encountered in orchard scenes.

1.3 Ablation Study on the CSDU Module

To validate the proposed CSDU module for lightweight upsampling and detail-preserving feature reconstruction, we compared it with several mainstream upsampling methods and further analyzed the contribution of its internal components. The results are presented in Table **8**.

Table 8. Ablation study of CSDU on the Orchard Pear dataset

Model Configuration	DWC conv	QCSM	GCT	PWC conv	mAP@50	mAP@50:95	Param(M)	GFLOPs	FPS
YOLOv11n Baseline (nn.Upsample)	×	×	×	×	89.00	47.50	2.60	6.30	218
Bilinear Interpolation	×	×	×	×	89.10	47.60	2.60	6.30	212
Transposed Conv (ConvTranspose2d)	×	×	×	×	89.80	48.40	2.89	7.20	186
PixelShuffle (Sub-Pixel Conv)	×	×	×	×	89.50	48.10	2.82	6.98	192
CARAFE	×	×	×	×	90.10	48.80	2.78	7.12	178
Upsample + DWConv only	✓	×	×	×	89.80	48.30	2.62	6.42	210
Upsample + DWConv + PWConv	✓	×	×	✓	90.30	49.10	2.65	6.58	207
Upsample + DWConv + GCT + PWConv	✓	×	✓	✓	90.80	49.70	2.65	6.58	207
CSDU (Full: DWConv+GCT+QCSM+PWConv)	✓	✓	✓	✓	91.90	51.40	2.70	6.80	201
CSDU w/ QCSM shift=2	✓	s=2	✓	✓	91.50	50.80	2.70	6.80	201
CSDU w/ QCSM shift=3	✓	s=3	✓	✓	91.20	50.30	2.70	6.80	201
CSDU w/ Standard Shuffle (no QCSM)	✓	Std.	✓	✓	90.60	49.50	2.70	6.80	203

Note: In the QCSM column, s=2/s=3 denotes the shift step variants, while Std. indicates the use of standard channel rearrangement only.

Among the standard baselines, nearest-neighbor interpolation achieves 89.0% mAP@50, while bilinear interpolation provides only a marginal improvement of 0.1%. Although transposed convolution and CARAFE further improve detection accuracy, they also introduce noticeable increases in model complexity. Specifically, transposed convolution increases the parameter count by approximately 11%, while CARAFE increases computational cost by about 13% GFLOPs relative to the baseline.

In contrast, the proposed CSDU achieves steady performance gains through a lightweight design. Adding depthwise convolution alone improves mAP@50 by 0.8%, indicating that per-channel spatial refinement is beneficial after interpolation-based upsampling. Introducing pointwise convolution further contributes 0.5%, highlighting the importance of channel projection in restoring feature interactions. Adding GCT brings another 0.5% improvement by enhancing inter-group channel reorganization. Most notably, adding QCSM yields a further 1.1% mAP@50 gain over the GCT-equipped variant (90.8% $\rightarrow$ 91.9%), showing that parameter-free directional cyclic shifts effectively strengthen cross-channel spatial interaction during upsampling.

As a result, the complete CSDU reaches 91.9% mAP@50, corresponding to a total gain of 2.9% over the baseline. We further evaluated the effect of the shift step in QCSM and found that the default setting s=1 gives the best overall performance. Larger shifts (s=2 and s=3)lead to gradual performance degradation, suggesting that excessive spatial displacement weakens local-detail alignment. Overall, these results indicate that CSDU effectively improves feature reconstruction quality during upsampling while maintaining low computational overhead.

1.4 Ablation Study on the MSCRB Module

To assess the effectiveness of the proposed MSCRB module in improving multi-scale representation and channel interaction, we conducted progressive ablation experiments on its key components, including multi-branch depthwise convolution, grouped channel transposition, quadrant channel-spatial mixing, residual connection, and channel expansion ratio. The results are listed in Table **9**.

Table 9. Ablation study of MSCRB on the Orchard Pear dataset

Model Configuration	MSDC	GCT	QCSM	Residual	mAP@50	mAP@50:95	Param(M)	GFLOPs	FPS
YOLOv11n Baseline (C3k2 in Neck)	×	×	×	×	89.00	47.50	2.60	6.30	218
Single DWConv k=3	×	×	×	×	89.30	47.80	2.54	6.18	215
MSDC (k={1,3,5})	✓	×	×	×	90.40	48.90	2.58	6.35	211
MSDC + GCT	✓	✓	×	×	90.90	49.40	2.58	6.35	210
MSDC + QCSM (w/o GCT)	✓	×	✓	×	90.70	49.10	2.58	6.35	210
Full MSCRB (MSDC+GCT+QCSM)	✓	✓	✓	×	91.20	49.90	2.62	6.42	208
MSCRB + Residual (r=2, default)	✓	✓	✓	✓	91.60	50.40	2.65	6.48	206
MSCRB r=1 (no expansion)	✓	✓	✓	✓	90.80	49.50	2.56	6.28	212
MSCRB r=3	✓	✓	✓	✓	91.30	50.10	2.78	6.72	196
MSCRB k={3,5} (2-branch)	✓	✓	✓	✓	91.20	49.90	2.63	6.44	208

Note: MSDC denotes the Multi-Scale parallel Depthwise Separable Convolution group. GCT stands for Grouped Channel Transpose (parameter-free). QCSM represents the Quadrant Channel-Spatial Mixer (parameter-free). r is the channel expansion factor (default r=2), k indicates the set of kernel sizes for the parallel depthwise separable convolutions (default k={1,3,5}).

Replacing the original neck block with a single 3×3 depthwise convolution improves mAP@50 by only 0.3% over the baseline, indicating that a single receptive field is insufficient for reliable fruit detection in complex orchard scenes. In contrast, the proposed multi-branch depthwise convolution with kernel sizes $\{1, 3, 5\}$ raises mAP@50 to 90.4%, corresponding to a gain of 1.4%. This result highlights the importance of integrating multiple receptive fields when dealing with fruits of different sizes and partial visibility conditions.

Building upon this design, adding GCT alone yields an additional 0.5% improvement, while adding QCSM alone provides a 0.3% gain. When both are jointly applied, the complete MSCRB improves mAP@50 to 91.2%. After further incorporating the residual connection, the performance reaches 91.6% mAP@50 and 50.4% mAP@50:95, corresponding to gains of 2.6% and 2.9% over the baseline, respectively. These results indicate that channel reorganization and directional spatial mixing are complementary, while the residual pathway helps stabilize optimization and feature propagation.

We also examined the effect of the channel expansion ratio r. The default setting r=2 yields the best performance, whereas both r=1 and r=3 perform worse. This observation reflects the typical trade-off between representational capacity and lightweight efficiency: insufficient expansion limits feature diversity, while excessive expansion introduces redundant parameters and slightly weakens generalization. Therefore, r=2 provides the most balanced configuration for the proposed module.

1.5 Ablation Study on the SOA-IoU Loss Function

To evaluate the effectiveness of the proposed SOA-IoU loss in handling scale variation, shape distortion, and gradient imbalance during box regression, we conducted systematic ablation experiments on its three core components: adaptive inner-box overlap, occlusion-aware shape regularization, and scale-aware dynamic focusing. The results are shown in Table **10**, and the hyperparameter sensitivity analysis is reported in Table **11**.

Compared with the baseline CIoU loss, replacing the fixed-ratio inner-box formulation with the proposed adaptive inner-box strategy improves mAP@50:95 from 47.90% to 48.20%. This result suggests that scale-adaptive regulation better stabilizes overlap supervision across objects of different sizes. For the

Table 10. Ablation study of SOA-IoU on the Orchard Pear dataset

Model Configuration	IoU_{inner}	Ω_{shape}	ϕ_i	P	R	mAP@50	mAP@50:95	AP_S	AP_M	AP_L	ΔmAP@50:95
YOLOv11n (Baseline)											
No.1 CIoU	×	×	×	75.90	97.00	89.00	47.50	28.30	49.20	56.80	—
Individual mechanism and design choice validation											
No.2	Fixed ratio(λ=0.7)	×	×	75.80	97.20	89.50	47.90	29.80	49.50	56.70	+0.40
No.3	✓ Adaptive	×	×	76.00	97.30	89.80	48.20	30.60	49.80	57.10	+0.70
No.4	×	Only ω_{wh}	×	75.85	97.10	89.40	47.90	28.50	49.90	57.20	+0.40
No.5	×	Only ω_{prior}	×	75.80	97.10	89.30	47.80	28.40	49.60	57.00	+0.30
No.6	×	✓ Full	×	75.95	97.20	89.60	48.10	28.70	50.10	57.50	+0.60
No.7	×	×	w/o scale prior	75.85	97.20	89.40	47.90	29.30	49.40	56.90	+0.40
No.8	×	×	w/ scale prior	75.90	97.30	89.60	48.10	30.00	49.50	57.00	+0.60
Pairwise combinations											
No.9	✓ Adaptive	✓ Full	×	76.05	97.60	90.30	48.60	30.90	50.40	57.60	+1.10
No.10	✓ Adaptive	×	w/ scale prior	76.00	97.50	90.20	48.50	31.20	50.00	57.20	+1.00
No.11	×	✓ Full	w/ scale prior	75.95	97.50	90.10	48.40	29.80	50.30	57.70	+0.90
Complete model											
SOA-IoU (Ours)	✓ Adaptive	✓ Full	w/ scale prior	76.12	98.00	91.20	49.15	31.80	50.90	58.10	+1.65

Note: Fixed ($\lambda = 0.7$) adopts the optimal fixed scaling ratio reported in Inner-IoU. "Only ω_{wh}" and "Only ω_{prior}" denote the shape deviation factor and the geometric prior regularization factor within the shape penalty term, respectively. "w/o scale prior" refers to assigning gradient weights based solely on sample outlierness, whereas "w/ scale prior" represents the complete gradient focusing mechanism proposed in this paper, which incorporates the scale-adaptive offset factor ϕ_i. AP_S, AP_M, and AP_L indicate the mAP@50:95 for small, medium, and large objects, respectively. ΔmAP@50:95 represents the increment relative to the baseline.

Table 11. Hyperparameter sensitivity analysis of SOA-IoU on the Orchard Pear dataset

Experiment Group	Setting	mAP@50	mAP@50:95	AP_S	AP_M	AP_L	ΔmAP@50:95
CIoU Baseline	—	89.00	47.50	28.30	49.20	56.80	—
SOA-IoU (default)	See below	91.20	49.15	31.80	50.90	58.10	+1.65
(a) Sensitivity to adaptive inner box scale range $[\lambda_{\min}, \lambda_{\max}]$ *(β=5, $\theta_1 : \theta_2$=2:1 fixed)*							
$[\lambda_{\min}, \lambda_{\max}]$	[0.4, 0.9]	90.50	48.40	30.80	50.30	57.20	+0.90
	[0.5, 1.0]*	91.20	49.15	31.80	50.90	58.10	+1.65
	[0.6, 1.0]	91.00	48.95	31.30	50.80	58.00	+1.45
	[0.7, 1.0]	90.60	48.55	30.20	50.50	57.90	+1.05
(b) Sensitivity to Sigmoid steepness β ($[\lambda_{\min}, \lambda_{\max}]$=[0.5,1.0], $\theta_1 : \theta_2$=2:1 fixed)							
β (Sigmoid steepness)	$\beta = 3$	90.80	48.70	30.90	50.60	57.90	+1.20
	$\beta = 5$*	91.20	49.15	31.80	50.90	58.10	+1.65
	$\beta = 8$	91.10	49.00	31.60	50.85	58.00	+1.50
	$\beta = 10$	90.70	48.60	31.10	50.50	57.70	+1.10
(c) Sensitivity to shape penalty weight ratio $\theta_1 : \theta_2$ ($[\lambda_{\min}, \lambda_{\max}]$=[0.5,1.0], $\beta = 5$ fixed)							
$\theta_1 : \theta_2$ ratio	$1 : 0$ (only ω_{wh})	90.60	48.55	30.50	50.60	57.80	+1.05
	$0 : 1$ (only ω_{prior})	90.40	48.35	29.90	50.30	57.60	+0.85
	$1 : 1$	90.90	48.85	31.20	50.70	57.90	+1.35
	$2 : 1$*	91.20	49.15	31.80	50.90	58.10	+1.65
	$1 : 2$	90.80	48.70	31.00	50.50	57.80	+1.20
	$3 : 1$	91.05	49.00	31.50	50.80	58.00	+1.50
(d) Sensitivity to gradient focusing scale offset α_0 ($[\lambda_{\min}, \lambda_{\max}]$=[0.5,1.0], $\beta = 5$, $\theta_1 : \theta_2$=2:1 fixed)							
α_0 (gradient focus base)	$\alpha_0 = 1.0$	90.70	48.60	30.60	50.60	57.90	+1.10
	$\alpha_0 = 1.5$*	91.20	49.15	31.80	50.90	58.10	+1.65
	$\alpha_0 = 2.0$	91.10	49.05	31.90	50.90	57.80	+1.55
	$\alpha_0 = 2.5$	90.80	48.70	31.50	50.50	57.40	+1.20

Note: The asterisk (*) denotes the default configuration ($[\lambda_{\min}, \lambda_{\max}] = [0.5, 1.0]$, $\beta = 5$, $\theta_1 : \theta_2 = 2 : 1$, $\alpha_0 = 1.5$). In each group of experiments, only the target hyperparameter is varied, while the remaining hyperparameters are fixed at their default values. The green-shaded row indicates the optimal configuration. ΔmAP@50:95 represents the increment relative to the CIoU baseline.

shape term, the complete shape formulation performs better than using either width-height consistency

or geometric-prior regularization alone, indicating that explicit size alignment and prior-guided shape constraints are complementary under partial occlusion.

In addition, introducing the scale-aware focusing mechanism further improves performance, particularly on small objects, showing that adaptive gradient reallocation is beneficial for hard regression samples. When any two components are combined, the performance consistently exceeds that of the individual components, demonstrating clear complementarity among the three designs.

The complete SOA-IoU achieves 91.20% mAP@50 and 49.15% mAP@50:95, corresponding to gains of 2.20% and 1.65% over the baseline, respectively. The improvements are also evident on scale-specific metrics, where APS, APM, and APL increase by 3.5%, 1.7%, and 1.3%, respectively. The largest gain on APS indicates that SOA-IoU is particularly effective for small fruits, which are the most challenging instances in orchard detection.

To further assess robustness to hyperparameter selection, we conducted controlled sensitivity analysis on several key settings. The results show that the default configuration consistently yields the best overall performance, while the variation range of mAP@50:95 across tested settings remains limited. This observation suggests that the performance improvement of SOA-IoU does not rely on narrow hyperparameter tuning, but rather reflects the robustness of the overall regression design.

1.6 Comparison of Loss Functions

To further validate the proposed SOA-IoU loss from the optimization perspective, we compared it with the strongest recent IoU-based loss functions, including CIoU, EIoU, Focal-EIoU, NWD, SIoU, and Wise-IoU. The results on the Orchard Pear dataset are listed in Table **12**. Compared with the baseline CIoU loss, SOA-IoU improves AP, AP50, and AP75 by 1.65%, 2.20%, and 2.00%, respectively. The consistent gains across different localization thresholds indicate that the proposed loss enhances not only coarse overlap alignment but also more precise boundary regression.

Table 12. Comparison of the proposed SOA-IoU with six mainstream IoU loss variants on the Orchard Pear dataset

Loss Function	AP	AP_{50}	AP_{75}	AP_s	AP_M	AP_L	Dense	Occluded
CIoU (Baseline)	47.50	89.00	49.80	28.30	49.20	56.80	43.80	41.20
EIoU	47.80	89.30	50.10	28.80	49.50	57.00	44.10	41.60
Focal-EIoU	48.00	89.50	50.40	29.10	49.70	57.20	44.40	41.90
NWD	47.60	88.80	49.50	29.50	48.90	55.80	43.50	41.50
SIoU	47.90	89.40	50.20	28.60	49.60	57.10	44.20	41.70
Wise-IoU	48.20	89.60	50.50	29.80	49.80	57.30	44.50	42.10
**SOA-IoU (Ours)**	**49.15**	**91.20**	**51.80**	**31.80**	**50.90**	**58.10**	**46.30**	**43.80**
Improvement vs. Baseline	**+1.65**	**+2.20**	**+2.00**	**+3.50**	**+1.70**	**+1.30**	**+2.50**	**+2.60**

The scale-wise results further highlight its advantage. Specifically, SOA-IoU improves AP_S, AP_M, and APL_{over} CIoU by 3.5%, 1.7%, and 1.3%, respectively. The largest improvement on small objects suggests that adaptive inner-box regulation is particularly helpful in stabilizing supervision under small localization deviations.

In orchard-specific challenging scenarios, SOA-IoU also performs favorably. On dense and occluded subsets, it improves performance over CIoU by 2.5% and 2.6%, respectively. These results indicate that the combination of shape-aware regularization and scale-aware dynamic focusing is well suited to the regression characteristics of orchard scenes, where overlap, occlusion, and ambiguous boundaries frequently occur together.

Compared with other advanced variants such as Wise-IoU and Focal-EIoU, SOA-IoU still achieves the best overall performance. This suggests that jointly considering scale variation, occlusion-induced shape distortion, and hard-sample gradient imbalance is more effective than optimizing only a single aspect of the regression process.

1.7 Cross-Dataset Generalization Ablation Study

To investigate whether the benefits of the proposed framework generalize beyond the self-constructed Orchard Pear dataset, we further conducted cross-dataset ablation experiments on Minne Apple and Mango. The detailed results are reported in Table **13**.

Table 13. Cross-dataset generalization ablation study on the Minne Apple and Mango datasets

Dataset	Baseline	Components				Detection Performance (%)				Δ	Speed
	YOLOv11n	CSP-SGLFE	SGEAE	CSDU	SOA-IoU	P	R	mAP @50	mAP @50:95	mAP @50	FPS
Minne Apple	✓	×	×	×	×	**96.20**	**86.00**	**60.20**	**33.50**	—	**212**
	✓	✓	×	×	×	97.80	87.00	63.50	34.70	+3.30	179
	✓	×	✓	×	×	95.60	85.50	62.10	34.20	+1.90	176
	✓	×	×	✓	×	96.40	85.80	61.50	34.60	+1.30	196
	✓	×	×	×	✓	97.15	86.20	61.70	34.51	+1.50	206
	✓	✓	✓	×	×	97.10	86.00	65.40	36.00	+5.20	167
	✓	✓	×	✓	✓	96.80	86.80	65.15	35.60	+4.95	170
	✓	×	✓	✓	✓	94.60	87.00	64.40	35.10	+4.20	163
	✓	✓	✓	✓	×	92.50	90.50	75.16	42.35	+14.96	159
	✓	✓	✓	✓	✓	**90.10**	**93.00**	**77.90**	**45.90**	**+17.70**	**156**
Mango	✓	×	×	×	×	**92.00**	**97.00**	**92.50**	**59.10**	—	**221**
	✓	✓	×	×	×	90.50	98.00	93.00	60.60	+0.50	187
	✓	×	✓	×	×	91.30	97.50	93.10	62.30	+0.60	185
	✓	×	×	✓	×	91.50	97.20	93.40	63.10	+0.90	204
	✓	×	×	×	✓	90.75	98.00	95.60	63.10	+3.10	214
	✓	✓	✓	×	×	91.10	99.00	94.60	62.00	+2.10	175
	✓	✓	×	✓	✓	92.20	98.50	95.25	63.10	+2.75	179
	✓	×	✓	✓	✓	91.25	96.00	95.40	64.00	+2.90	171
	✓	✓	✓	✓	×	93.50	98.80	97.10	67.15	+4.60	168
	✓	✓	✓	✓	✓	**93.90**	**99.00**	**98.20**	**68.60**	**+5.70**	**165**

2 RESULTS AND ANALYSES

2.1 Grad-CAM Visualization of the Overall Ablation Study

To provide an intuitive interpretation of how different components progressively improve feature representation, we further performed Grad-CAM visualization for multiple ablation configurations on the Orchard Pear dataset. The results are shown in Figure **13**.

The baseline model exhibits relatively dispersed activation over background regions such as branches and leaves, indicating limited target selectivity in the presence of dense occlusion and small-object interference.

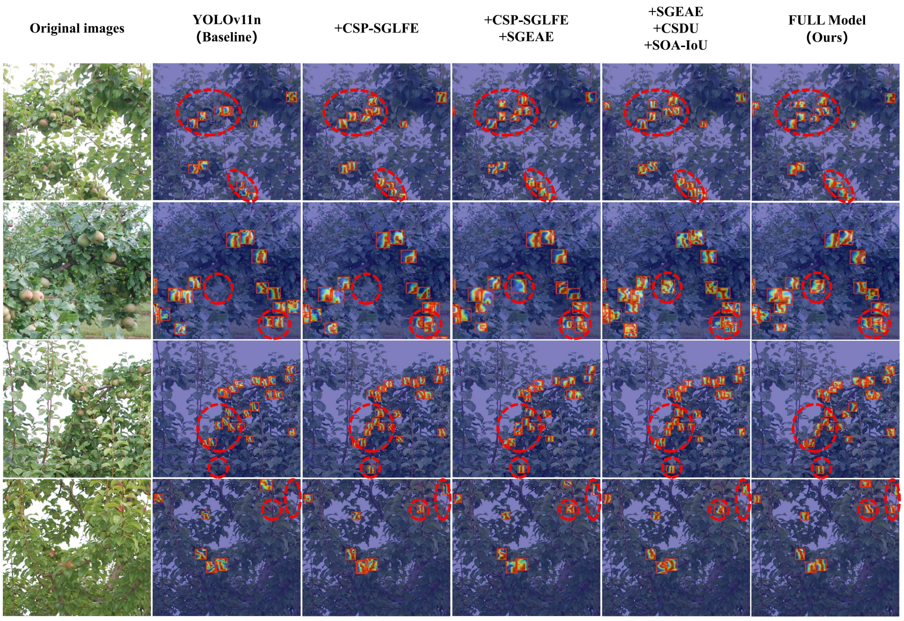

Figure 13. Grad-CAM visualization of ablation configurations on the Orchard Pear dataset

After introducing CSP-SGLFE, the activation becomes more concentrated around fruit regions, suggesting that global-local context modeling helps the detector focus on more complete target cues. When SGEAE is further incorporated, the responses become more localized on the visible regions of partially occluded fruits, reflecting the benefit of adaptive sparse transformation for handling degraded local appearances.

After adding CSDU and SOA-IoU, the activation intensity on small fruits becomes noticeably stronger. This observation suggests that lightweight channel-spatial fusion and scale-aware regression optimization jointly improve discriminability for difficult targets. The full model produces the most compact and consistent target-focused responses while substantially suppressing background interference. These visual results are well aligned with the quantitative ablation findings and further support the effectiveness of the proposed framework.

2.2 Failure Cases Analysis

While the proposed framework demonstrates robust performance in complex unstructured orchards, difficult cases provide a more revealing lens for assessing its behavior. Figure **14** shows head-to-head detection comparisons between the YOLOv11n baseline and the proposed method on representative hard examples across the three datasets. In the cases shown, the baseline misses small or partially occluded fruits that the proposed method successfully recovers, with confidence scores rising from 0.72 to 0.77 in a representative pear instance. Red dashed boxes mark missed detections by the baseline. These examples complement the scenario-wise quantitative results in Section 3.3 by isolating individual hard instances that the aggregate metrics average over.

Despite these improvements, certain extreme cases remain beyond the reach of the proposed method. Figure **15** illustrates typical failure cases, which primarily manifest as missed detections (highlighted by red dashed boxes).

These failures generally occur under three extreme conditions: (1) Severe structural destruction caused by thick foreground branches (Row 1), leaving insufficient visible areas for feature extraction; (2) Extreme

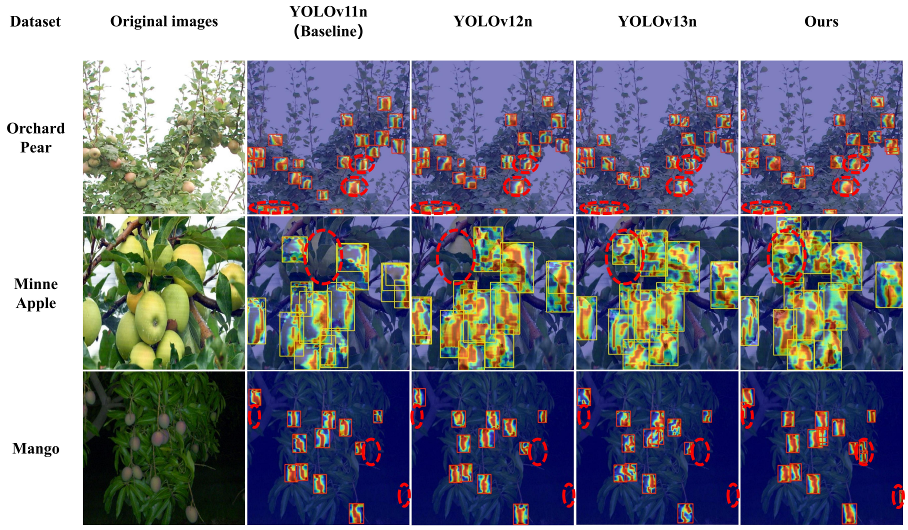

Figure 14. Detection comparison on difficult cases across three datasets. Each row shows an input image (left), the baseline YOLOv11n result (middle), and the proposed method's result (right). Yellow zoomed insets highlight regions of interest; red dashed boxes indicate missed detections produced by the baseline. The proposed method recovers fruits that the baseline omits, with confidence scores illustrated on representative instances.

scale degradation of miniature targets in the deep background (Row 2), where spatial resolution and texture details are almost entirely lost; and (3) Severe color camouflage (Row 3), where fruits perfectly blend into the surrounding canopy under complex shadows, neutralizing local contrast.

The fundamental reason for these limitations is that our method, despite its enhanced selective information propagation, still relies entirely on 2D RGB visual cues. When appearance features are irreversibly degraded, the network struggles to construct reliable semantic representations. Addressing these extreme edge cases by incorporating multi-modal sensing (e.g., depth or thermal imaging) to provide complementary geometric or physical priors remains an important direction for future research.

2.3 Statistical Significance Verification

To rigorously exclude the influence of training randomness on the reported conclusions, we performed repeated independent runs for the proposed method and the main comparison models. For each paired comparison, the complete training and evaluation procedure was repeated using 10 independent random seeds, namely $\{0, 7, 42, 88, 123, 256, 314, 421, 512, 777\}$.

All other experimental settings, including the data split, augmentation strategy, and hyperparameters, were kept identical to those used in the main experiments, so that only the random initialization state was changed. For each run, mAP@50 and mAP@50:95 on the test set were recorded.

Based on the 10-run results, we computed the mean and standard deviation for each model and further conducted paired hypothesis testing. Considering the relatively limited sample size $n = 10$ and the paired dependency across runs, we report two complementary statistical tests: (1) the Wilcoxon signed-rank test, which is non-parametric, robust to outliers, and does not assume normality; and (2) the paired-sample t-test, which is parametric and provides higher statistical power when approximate normality holds. For both tests, the null hypothesis H_0 states that there is no significant difference between the proposed method and the compared model in terms of mAP@50:95, and the significance level was set to $\alpha = 0.05$.

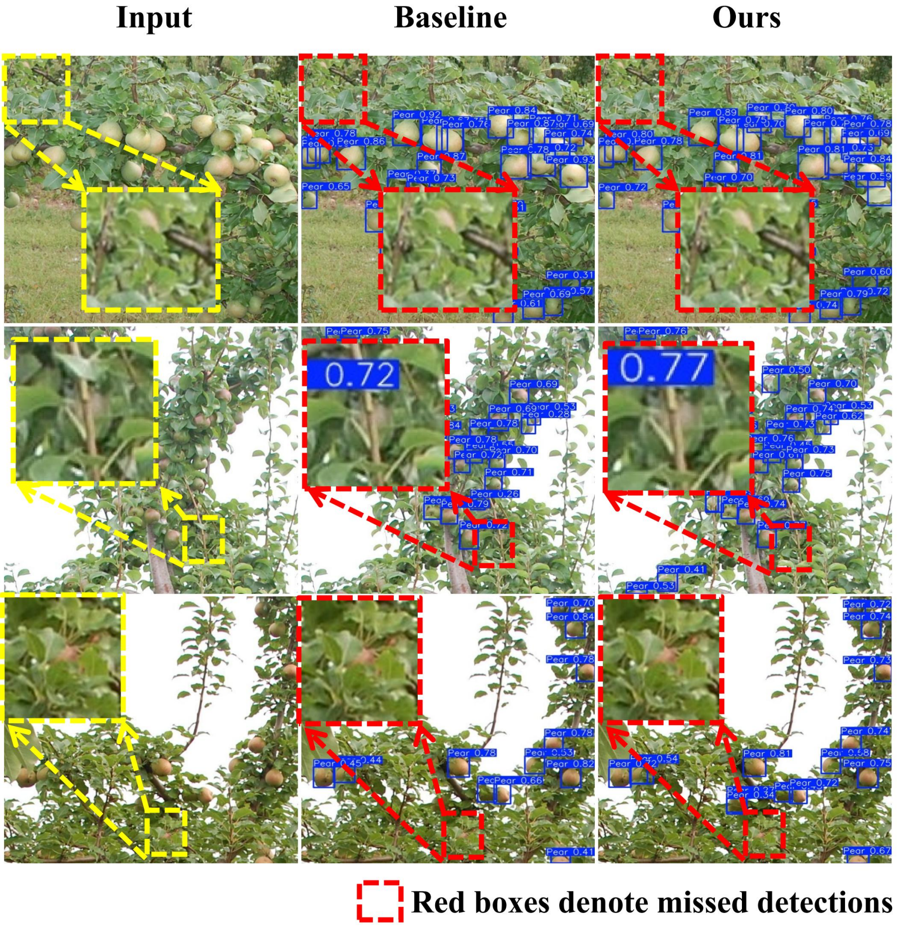

Figure 15. Visualization of typical failure cases in extreme orchard scenarios

The results are summarized in Table **14**. Across all six paired comparisons, the Wilcoxon p-values are 0.0020, and the paired t-test p-values are all smaller than 10^{-5}. Both tests therefore consistently reject the null hypothesis, even under the stricter significance level of $\alpha = 0.01$. These findings indicate that the superiority of the proposed method is not attributable to random fluctuations, but reflects a statistically robust performance improvement.

In addition to achieving the best mean accuracy, the proposed method also exhibits the lowest performance variance among all compared models, with a standard deviation of ± 0.20 for mAP@50 and ± 0.17 for mAP@50:95. Beyond the accuracy gains, this reduced variance indicates that the proposed selective information propagation framework also yields more stable optimization and convergence behavior. Such stability is particularly valuable for lightweight orchard detection, where small models are often more sensitive to training perturbations and data variation.

Overall, the statistical analysis confirms that the gains reported in the previous sections are both significant and reproducible, and strengthens the reliability of the reported results.

Table 14. Statistical significance verification of the proposed method against six comparison models on the Orchard Pear dataset

Model	mAP@50 (Mean±Std)	mAP@50:95 (Mean±Std)	Wilcoxon p-value	Paired t-test p-value	Sig.
YOLOv11n	88.94 ± 0.32	47.43 ± 0.25	0.0020**	3.8×10^{-8} **	✓
YOLOv12n	89.76 ± 0.28	48.24 ± 0.22	0.0020**	5.1×10^{-8} **	✓
YOLOv13n	90.45 ± 0.26	49.06 ± 0.20	0.0020**	2.7×10^{-7} **	✓
Rose-Mamba-YOLO	91.24 ± 0.30	49.74 ± 0.27	0.0020**	8.3×10^{-7} **	✓
VM-YOLO	90.76 ± 0.25	49.38 ± 0.21	0.0020**	6.2×10^{-7} **	✓
YOLO-CSB	91.55 ± 0.27	50.16 ± 0.23	0.0020**	1.5×10^{-6} **	✓
**Ours**	**95.20±0.20**	**54.60±0.17**	—	—	—

*Note: ** denotes statistical significance at the $\alpha = 0.01$ level. With $n = 10$, the minimum attainable two-sided p-value for the Wilcoxon signed-rank test is $2/2^{10} \approx 0.00195$. The value $p = 0.0020$ reported in the table corresponds to this theoretical lower bound, indicating that the proposed method consistently outperforms the compared methods across all 10 runs. The paired t-test has 9 degrees of freedom (df = 9). Mean±Std is derived from 10 independent runs. All compared methods are evaluated using the same set of 10 random seeds.*

3 COMPONENT-LEVEL COMPARISON WITH LIGHTWEIGHT BACKBONES, MODULES, AND AN AGRICULTURE-SPECIFIC DETECTOR

While the state-of-the-art comparison reported in the main text evaluates our method against full end-to-end detectors, an important question remains unanswered: is the observed performance gain driven by the coordinated operation of the four proposed modules, or could it be attributed to a favorable choice of lightweight building blocks alone? To answer this question, we designed a set of controlled component-replacement experiments in which either the full backbone or a single internal module of YOLOv11n is substituted with a representative lightweight counterpart, while the rest of the detection pipeline—neck, head, loss, training protocol, input resolution (640×640), and data splits—remains identical to Section 3.1.1. This design isolates the contribution of each lightweight building block under a fair comparison protocol. We additionally include FruitDet, a representative agriculture-specific detector, as an independent end-to-end baseline. The six resulting variants span three architectural families that are most commonly considered when constructing lightweight detectors for agricultural deployment: mobile CNN backbones, mobile Transformer backbones, and agriculture-specific detectors.

Three observations arise from Table **15**. First, the strongest backbone replacement, EfficientFormerV2, is competitive with our method on raw accuracy—94.50% mAP@50 and 54.50% mAP@50:95, the latter within 0.10% of ours—so the relevant difference here is efficiency rather than accuracy. It reaches this level with almost twice the parameters (4.84 M vs. 2.56 M) and nearly twice the GFLOPs (10.20 vs. 5.60), whereas our method matches it at roughly half the cost, which is the property that matters under the embedded budget targeted in Section 3.7. MobileNetV4 and EfficientViT both reach 94.10% mAP@50, and for MobileNetV4 this comes at nearly $4\times$ the computation (21.00 GFLOPs vs. 5.60).

Second, module-level replacements (C3k2-MBConv from EfficientNet, C2PSA-CGA from EfficientViT) provide clear improvements over the YOLOv11n baseline (+4.90% and +5.10% mAP@50, respectively), but still fall short of our method by 1.30% and 1.10% mAP@50, even though C2PSA-CGA uses a comparable computational budget (6.30 GFLOPs) and a similar parameter count (2.56 M) to our method. This pattern indicates that replacing a single module with an efficient alternative is insufficient to resolve

Table 15. Extended comparison on the Orchard Pear dataset. Lightweight backbones and modules are integrated into the YOLOv11n pipeline under a unified training protocol for fair comparison; FruitDet is included as an independent agriculture-specific method.

Category	Variant	Replaced Component	GFLOPs	Params(M)	mAP@50	mAP@50:95	APs	FPS
Baseline	YOLOv11n (Jocher and Qiu, 2024)	—	6.30	2.60	89.00	47.50	28.30	218
Mobile CNN – backbone	MobileNetV4 (Qin et al., 2024)	Full backbone	21.00	5.43	94.10	53.30	37.23	124
Mobile CNN – module	C3k2-MBConv (Shang et al., 2023)	C3k2 (→ EfficientNet MBConv)	6.10	2.88	93.90	51.20	35.89	196
Mobile ViT – backbone	EfficientViT (Cai et al., 2023)	Full backbone	7.9	3.74	94.10	52.74	36.16	171
Mobile ViT – backbone	EfficientFormerV2 (Li et al., 2023)	Full backbone	10.20	4.84	94.50	54.50	37.64	127
Mobile ViT – module	C2PSA-CGA (Mehta and Rastegari, 2021)	C2PSA (→ Cascaded Group Attention)	6.30	2.56	94.10	53.60	37.49	208
Agriculture-specific	FruitDet (Kateb et al., 2021)	End-to-end replacement	6.79	1.46	92.40	51.13	30.65	194
—	**Ours**	—	**5.60**	**2.56**	**95.20**	**54.60**	**38.20**	**162**

the compound representation–transformation–fusion–optimization bottlenecks that our framework targets; isolated component substitutions improve only one stage of the pipeline at a time.

Third, FruitDet, despite being specifically designed for fruit detection, reaches only 92.40% mAP@50—2.80% below our method—and shows the largest drop on small objects (APs = 30.65%, compared with 38.20% for ours). This suggests that general-purpose agricultural detection architectures, typically optimized for moderately occluded fruit categories such as apples, do not automatically transfer to the more compound challenge posed by dense small-fruit detection under heavy foliage-similar coloring and clustered spurs.

These results answer the question raised at the start of this appendix. No single backbone or module swap reaches our method's accuracy at a comparable cost: the alternatives either fall short on accuracy or, in the case of EfficientFormerV2, match it only with roughly twice the parameters and computation. Our method attains the same accuracy at the smallest computational footprint (5.60 GFLOPs) and one of the smallest parameter counts (2.56 M) in the comparison, which points to the coordinated operation of the four modules (Section 2) rather than to any single lightweight building block. For completeness, we note that AgriDet, sometimes listed alongside FruitDet in agricultural-detection surveys, is a plant-leaf-disease severity classification framework based on INC-VGGN rather than an object detector, and is therefore not applicable as a direct baseline in this comparison.

4 QUANTITATIVE ATTENTION ALIGNMENT ANALYSIS

To support the qualitative cross-dataset Grad-CAM observations reported in the main text, we report the quantitative attention alignment metrics—Pointing Game accuracy and Attention-IoU—for each progressive configuration of the proposed framework in Table **16**. The computation protocol and interpretation are detailed in Section 3.3.

Table 16. Quantitative evaluation of Grad-CAM attention alignment with ground-truth object locations, averaged over the Orchard Pear test set. Higher is better for both metrics.

**Configuration**	**Pointing Game Acc. (%)**	**A-IoU**
YOLOv11n (baseline)	71.30	0.342
+ CSP-SGLFE	76.20	0.401
+ CSP-SGLFE + SGEAE	79.80	0.448
+ CSP-SGLFE + SGEAE + CSDU + MSCRB	82.50	0.487
Full (+ SOA-IoU)	84.70	0.518
Relative improvement over baseline	+18.8%	+51.5%
